# March1-dependent modulation of donor MHC II on CD103^+^ dendritic cells mitigates alloimmunity

**DOI:** 10.1038/s41467-018-05572-z

**Published:** 2018-08-28

**Authors:** Thiago J. Borges, Naoka Murakami, Felipe D. Machado, Ayesha Murshid, Benjamin J. Lang, Rafael L. Lopes, Laura M. Bellan, Mayuko Uehara, Krist H. Antunes, Maria José Pérez-Saéz, Gabriel Birrane, Priscila Vianna, João Ismael B. Gonçalves, Rafael F. Zanin, Jamil Azzi, Reza Abdi, Satoshi Ishido, Jeoung-Sook Shin, Ana Paula D. Souza, Stuart K. Calderwood, Leonardo V. Riella, Cristina Bonorino

**Affiliations:** 1 School of Biosciences and Biomedical Research Institute, Pontifícia Universidade Católica do Rio Grande do Sul, PUCRS. Av. Ipiranga, 6690, IPB, 2nd floor, lab 6, Porto Alegre, RS Brazil; 2 Schuster Family Transplantation Research Center, Renal Division, Brigham and Women’s Hospital, Harvard Medical School, 221 Longwood Ave, Boston, MA 02115 USA; 3 Department of Radiation Oncology, Beth Israel Deaconess Medical Center, Harvard Medical School, 330 Brookline Ave, Boston, MA 02215 USA; 4 School of Pharmacy and Centro Infant, Biomedical Research Institute, PUCRS. Av. Ipiranga, 6690, IPB, 2nd floor, lab 31, Porto Alegre, RS Brazil; 5 Department of Medicine, Beth Israel Deaconess Medical Center, Harvard Medical School, 99 Brookline Ave, Boston, MA 02215 USA; 6 Genetics Department, Universidade Federal do Rio Grande do Sul, Av. Bento Gonçalves, 9500 Porto Alegre, RS Brazil; 7 Department of Microbiology, Hyogo College of Medicine, Nishinomiya, Hyogo 663-8501 Japan; 8 Department of Microbiology and Immunology, Sandler Asthma Basic Research Center, University of California San Francisco, 513 Parnassus Ave, HSE-201, San Francisco, CA 94143-0414 USA; 9 Present Address: Department of Health and Human Development, La Salle University, Av. Victor Barreto, 2288 Canoas, RS Brazil; 10 Present Address: Department of Basic Health Sciences, Laboratory of Immunotherapy, Federal University of Health Sciences of Porto Alegre, Rua Sarmento Leite, 245 Porto Alegre, RS Brazil

## Abstract

In transplantation, donor dendritic cells (do-DCs) initiate the alloimmune response either by direct interaction with host T cells or by transferring intact donor MHC to host DCs. However, how do-DCs can be targeted for improving allograft survival is still unclear. Here we show CD103^+^ DCs are the major do-DC subset involved in the acute rejection of murine skin transplants. In the absence of CD103^+^ do-DCs, less donor MHC-II is carried to host lymph nodes, fewer allogenic T cells are primed and allograft survival is prolonged. Incubation of skin grafts with the anti-inflammatory mycobacterial protein DnaK reduces donor MHC-II on CD103^+^DCs and prolongs graft survival. This effect is mediated through IL-10-induced March1, which ubiquitinates and decreases MHC-II levels. Importantly, in vitro pre-treatment of human DCs with DnaK reduces their ability to prime alloreactive T cells. Our findings demonstrate a novel therapeutic approach to dampen alloimmunity by targeting donor MHC-II on CD103^+^DCs.

## Abstract (web summary)

Donor-derived dendritic cells (do-DC) in the graft can contribute to the induction of alloimmunity and tissue rejection, but how do-DC can be targeted for improving graft survival is unclear. Here the authors show that reducing MHC-II expression on do-DCs by DnaK pre-treatment can decrease the priming of alloimmunity and prolong graft survival in mouse models.

## Introduction

Dendritic cells (DCs) initiate adaptive immune responses by delivering the prerequisite signals for specific T cell activation. DCs present peptides in MHC class II (MHC II) and I cell surface complexes and, when activated, provide costimulatory signaling (i.e., CD86) and cytokines that modulate the type of T cell response that ensues^1–3^. The activation of CD4^+^ T cells upon interaction with MHC II-peptide complexes on DCs is the key event in generating protective immune responses to infection, as well as detrimental autoimmune, allergic, and alloreactive responses. In alloimmune responses, draining lymph nodes (dLN) serve as the optimal site to prime anti-donor T cells by donor DCs carrying and transferring donor intact MHC molecules to host DCs via extracellular vesicles^4^. Mouse skin contains three major subsets of APCs including two dermal and one epidermal subset. Dermal DCs (DDCs) include CD103^+^ DCs (also known as cDCs1) and CD11b^+^ DCs (or cDCs2)^5^, while Langerhan’s cells (LCs) are in the epidermis. Although LCs share some characteristics of DC lineage, they are currently classified as macrophages^6,7^. Migratory DCs can also be found in the LN along with resident DC subsets, which include CD8a^+^ and CD8a^−^ DCs. In human skin, CD141^+^ and CD1c^+^ DDCs are the counterparts of murine CD103^+^ and CD11b^+^ DDCs, respectively^5^. However, in skin transplantation, the specific donor DC subsets, migrating to dLN and transferring donor MHC antigens to host DCs have not been determined.

Current strategies to prevent graft rejection are largely based on the use of drugs that inhibit non-specific T cell activation and proliferation^8^, while more recent strategies have also targeted costimulatory molecules^9^. These therapies have been undoubtedly useful for better clinical results, however the overall outcome of such approaches directed at undesired T cell responses is challenged by off-target side effects. We hypothesized that a strategy to target donor DCs, through the modulation of donor MHC antigens, constitutes an important complementary therapeutic approach. However, to achieve this goal, it is first crucial to identify the leading donor DC subsets responsible for the alloreactive priming.

Tolerogenic DCs have been characterized by the low expression of MHC and costimulatory molecules. As previously reported by our group and others, DnaK, the bacterial ortholog of murine heat shock protein (Hsp)a1a gene product (Hsp70), can modulate MHC II expression and IL-10 production on DCs^10,11^. It also has anti-inflammatory effects in models of autoimmunity, like arthritis^12,13^. Furthermore, membrane-associated RING-CH 1 (March1) is an E3 ubiquitin ligase that ubiquitinates a conserved lysine residue in the cytoplasmic tail of the MHC II-β chain^14,15^. Induction of March1 is driven by interleukin IL-10^16^ and leads to ubiquitination of MHC II and CD86, resulting in lysosomal degradation and decreased surface expression of these proteins^17^. Whether targeting March1 could promote tolerogenic DCs and prolong graft survival has not been tested.

In the present study, we have identified that skin-migrating CD103^+^ DCs are the major DC subset carrying donor MHC molecules. These cells have a critical role in shuttling donor MHC to the allograft dLNs and transferring donor MHC to host DCs, which is required for an efficient priming of donor-reactive T cells. In addition, Batf3^−/−^ skins (lacking CD103^+^ DCs) are less immunogenic and carry less allo-MHC II in the transplanted tissue. We next determined whether downregulation of donor MHC II expression within this DC subset could extend graft survival. The in situ treatment of donor skin grafts with DnaK prior to transplant induces IL-10 and decreases donor MHC II levels in CD103^+^ DCs, reducing alloreactive T cell priming and extending graft survival. We newly identify that DnaK is a strong inducer of March1. IL-10 induced by DnaK activates March1-mediated ubiquitination of MHC II and its subsequent MHC II degradation. In human DCs, DnaK also induces MARCH1 and downregulates HLA-DR (MHC II) levels in CD141^+^, but not CD1c^+^ DCs. We therefore propose that targeting donor CD103^+^/CD141^+^ DCs prior to transplant constitutes a novel approach to reduce immunogenicity of the transplanted allograft upon transplantation.

## Results

### CD103^+^ DCs is the major DC subset carrying donor MHC II

**Fig. 1:**
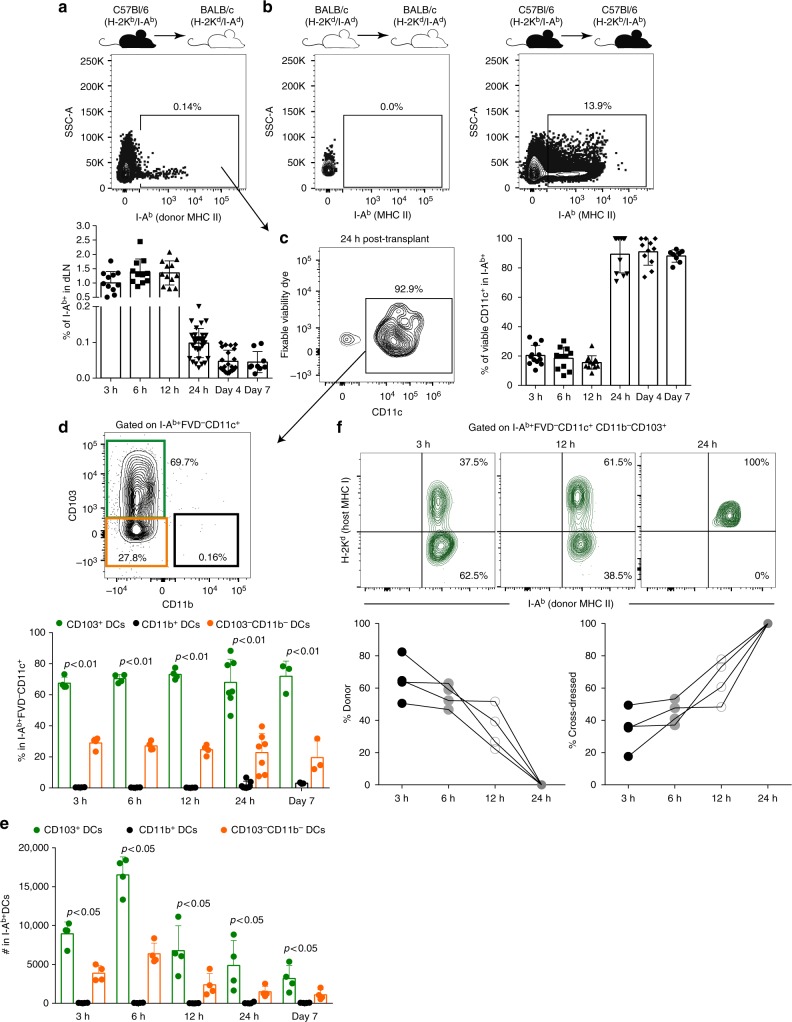
CD103^+^ DCs is the major subset carrying allo-MHC II. **a** Detection and quantification of I-A^b+^ events in total dLN cells (ungated) of BALB/c mice (H-2K^d^/I-A^d^) that received allogeneic B6 skin grafts (H-2K^b^/I-A^b^), 24 h post-transplant. **b** Detection of I-A^b+^ events in dLN of BALB/c mice (H-2K^d^/I-A^d^) that received syngeneic skin grafts (H-2K^d^/I-A^d^) or B6 mice (H-2K^b^/I-A^b^) that received syngeneic skin grafts (H-2K^b^/I-A^b^), 24 h post-transplant. **c** Representative contour plot and quantification of live (fixable viability dye^−^) DCs (CD11c^+^) cells in I-A^b+^ population in dLN of BALB/c mice (H-2K^d^/I-A^d^) that received allogeneic B6 skin grafts (H-2K^b^/I-A^b^) overtime. Results are pooled from eight experiments (*n* = 11–32 mice per time-point/group). **d** Phenotype and quantification of CD103^+^CD11b-, CD103^−^CD11b^+^ and CD103^−^CD11b^−^ DC subsets pre-gated on I-A^b+^FVD^−^CD11c^+^ population. Statistics using ANOVA with Tukey post-test (*n* = 3–7 mice per time-point/group). Results are pooled from two experiments. **e** Absolute numbers of I-A^b+^CD103^+^, I-A^b+^CD11b^+^ and I-A^b+^CD103^−^CD11b^−^ DCs. Bar graphs represent the mean ± S.D. Statistics using ANOVA with Tukey post-test (*n* = 3–4 mice per time-point/group). Representative results of two independent experiments. **f** Analysis of donor-derived (I-A^b+^H-2K^d−^) and host cross-dressed (I-A^b+^H-2K^d+^) cells in CD103^+^ DCs overtime. Each line represents one mouse. Representative results of two independent experiments

To determine which DC subset was carrying donor MHC antigens in dLN, we transplanted C57Bl/6 (B6, H-2K^b^/I-A^b^) skins into BALB/c (H-2K^d^/I-A^d^) hosts and tracked donor MHC II (I-A^b^) in host dLN using flow cytometry. We found that MHC II (I-A^b^) was present in dLN from 3 h to day 7 post-transplant (Fig. 1a). Importantly, we could not detect those antigens when BALB/c skins were transplanted into BALB/c hosts (both H-2K^d^/I-A^d^) (Fig. 1b) or when we transplanted MHC II^−/−^ skins (B6 background) into BALB/c hosts (Supplementary Fig. 1a), ensuring the specificity of our detection. In very early time-points (3–12 h post-transplant) around 15–20% of I-A^b+^ events expressed the DC marker CD11c, while from day 1 to 7 post-transplant, majority of the donor-derived MHC II was present in a CD11c^+^ population (Fig. 1c). We then analyzed the phenotype of DCs carrying donor MHC II molecules (I-A^b+^CD11c^+^ cells). Remarkably, the majority (~65%) of the I-A^b+^ DCs had the CD103^+^CD11b^−^ phenotype (Fig. 1d; Supplementary Fig. 2a) in all time-points analyzed. Absolute total numbers confirmed CD103^+^ DCs as the major DC subset carrying I-A^b^ (Fig. 1e), with these cells peaking at 6 h post-transplant. To address whether donor CD103^+^ DCs were important contributors of transferring donor antigens to host DCs, we tracked donor I-A^b^ in very early time-points (3 h, 6 h and 12 h) post-transplantation, before they die and cross-dress host DCs. We found that at 3 and 6 h post-transplant ~60% of CD103^+^ DCs carrying I-A^b^ did not express host MHC I (H-2K^d^) when reached dLN (Fig. 1f). However, at 24 h after the transplant, 100% of I-A^b+^ cells were also H-2K^d+^, suggesting that they were cross-dressed (Fig. 1f). Further detailed characterization revealed that I-A^b+^ DCs possessed a CD11c^+^CD207^+^CD11b^−^CD103^+^ phenotype (Supplementary Fig. 2b), confirming that they were skin migratory cells. Lastly, we validated that CD103^+^ DCs were the major subset carrying donor MHC II using a different tracking model, in which we transplanted skins from CD45.1 (H-2K^b^/I-A^b^) mice into CD45.2 BALB/c (H-2K^d^/I-A^d^) hosts (Supplementary Figs. 3 and 4).

### Batf3^−/−^ grafts carry less donor MHCII and are less immunogenic

**Fig. 2:**
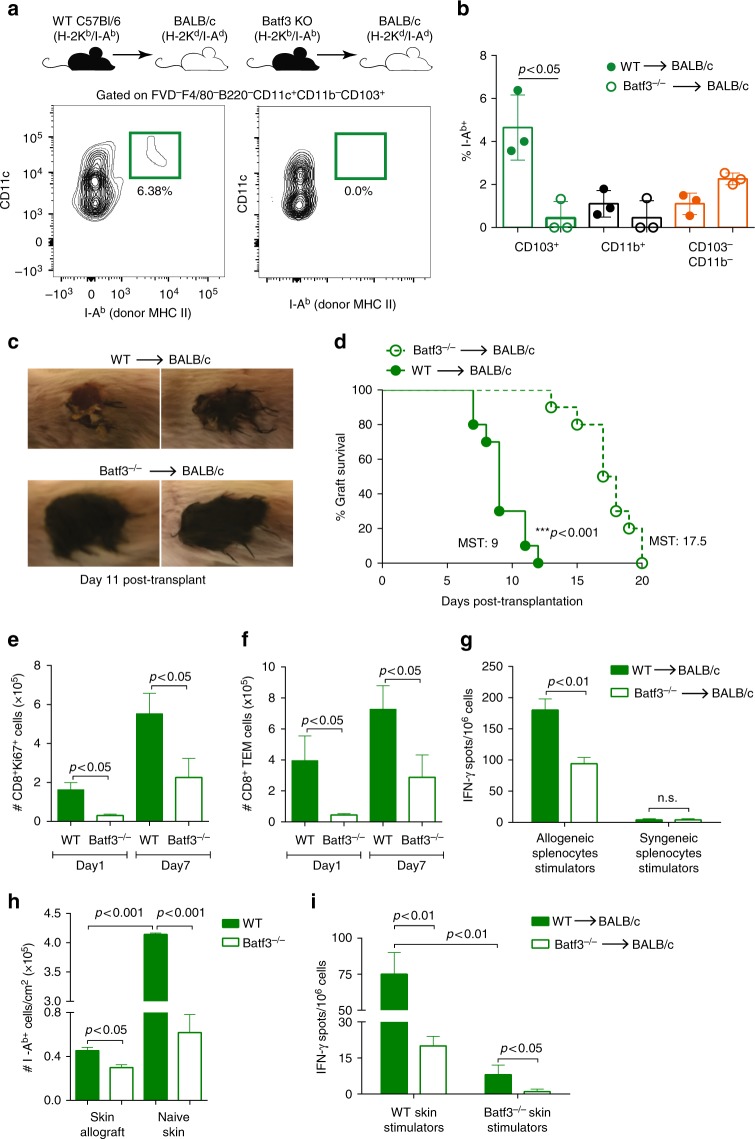
Batf3^−/−^ skins have better survival and induce decreased T cell-mediated allo-responses. Skin allografts from WT or Batf3^−/−^ mice (both H-2K^b^/I-A^b^) were transplanted into fully MHC-mismatched BALB/c (H-2K^d^/I-A^d^) recipients. **a** The presence of I-A^b+^ (donor MHC II) in dLN cross-dressed host CD103^+^ DCs was analyzed by flow cytometry, 24 h post-transplant. **b** Percentage of I-A^b+^ cells in cross-dressed host DC subsets of mice that received WT or Batf3^−/−^ skins, 24 h post-transplant. Bar graphs represent the mean ± S.D. Statistics using ANOVA with Tukey post-test. **c** Representative images of skin grafts at day 11 post-transplant. **d** Percent of graft survival. Mean survival time (MST). **p < 0.01 by log-rank test (*n* = 10 mice per group). Pooled from two independent experiments. **e** Numbers of CD8^+^Ki67^+^ and **f** CD8^+^ TEM cells from allograft dLN harvested 1 and 7 days post-transplant (*n* = 3–5 mice per time-point/group). Bar graphs represent the mean ± S.D. Statistics using ANOVA with Tukey post-test. **g** T cells from WT or Batf3^−/−^ allografts’ dLN were magnetically isolated (96 h post-transplant) and cultured with allogeneic (B6) or syngeneic (BALB/c) irradiated splenocytes for 48 h in mouse IFN-γ-coated plates. IFN-γ production by T cells was measured by ELISPOT. Numbers of spots per million T cells ± S.D. in triplicates and pooled from five mice. Statistics using *t* test. **h** Absolute number of total I-A^b+^ cells (gated on viable singlets) isolated from WT or Batf3^−/−^ skin allografts 24 h after the transplant or WT or Batf3^−/−^ naive skins. Bar graphs represent the mean ± S.D. Statistics using ANOVA with Tukey post-test. **i** T cells from WT or Batf3^−/−^ allografts’ dLN were magnetically isolated (96 h post-transplant) and cultured with skin cells isolated from WT or Batf3^−/−^ allografts 24 h after the transplant. Cells were cultured for 48 h in mouse IFN-γ-coated plates, and IFN-γ production by T cells was measured by ELISPOT. Numbers of spots per million T cells ± S.D. in triplicates and pooled from five mice. Statistics using *t* test. Representative results of at least two independent experiments

The CD103^+^ DC subset requires the transcription factor *Batf3* for their development in mice^18^. To confirm the role of donor CD103^+^ DCs in transporting donor MHC II to dLN, we transplanted skin grafts from Batf3^−/−^ donors, which lack CD103^+^ DCs, into BALB/c hosts (intact host CD103^+^ DCs). Host DCs cross-dressed with allo-MHC II were absent in dLN of mice that received Batf3^−/−^ allografts, 24 h after the transplant (Fig. 2a). We also observed a reduced percentage of total I-A^b+^ cells in dLN of Batf3^−/−^ graft recipients (Fig. 2b), supporting that CD103^+^ DCs is the dominant donor cell subset transferring donor MHC II molecules. Accordingly, mice that received Batf3^−/−^ skins presented increased allograft survival compared to WT skins, with an MST of 17.5 days compared with 9 days, respectively (*n* = 10 per group; *p* *<* 0.001) (Fig. 2c, d). Analysis of dLNs revealed a significant reduction in the numbers of proliferating (Ki67^+^) CD8^+^ T cells (Fig. 2e) and CD8^+^ T effector memory cells (TEM—CD8^+^CD44^+^CD62L^−^ cells; Fig. 2f). To test whether allogeneic T cells were efficiently primed in the absence of CD103^+^ DCs, we isolated dLN T cells from fully-MHC mismatched transplant recipients that had received either a WT or Batf3^−/−^ skins 96 h before. Those cells were exposed by direct allorecognition to irradiated allogeneic (B6) or syngeneic (BALB/c) splenic stimulator cells. The production of IFN-γ by dLN T cells was tested by enzyme-linked immunospot (ELISPOT). When T cells isolated from mice that received Batf3^−/−^ allografts were cultured with allogeneic stimulators, they produced significantly less IFN-γ compared to WT allografts (Fig. 2g). These data suggest that donor CD103^+^ DCs have a critical role in priming donor-specific host T cells.

We next assessed the possibility that Batf3^−/−^ skins contain less alloantigens, since they lack the main DC subset carrying I-A^b^ antigens. We quantified the numbers of I-A^b+^ cells in WT or Batf3^−/−^ naive skins, as well as WT or Batf3^−/−^ skin allografts, 24 h after transplantation. WT skin allografts presented significantly less I-A^b+^ cells when compared to WT naive skin (Fig. 2h), suggesting that I-A^b^-carrying cells were emigrating from the skin or dying quickly after transplantation, as previously reported^19^. Interestingly, Batf3^−/−^ naive skin carried remarkably less I-A^b+^ cells (~85% reduction) when compared to WT naive skin (Fig. 2h). The same was true when we compared Batf3^−/−^ skin grafts to WT grafts (Fig. 2h), revealing that less donor MHC II remained in the transplanted tissue.

We then hypothesized that allograft-derived Batf3^−/−^ skin cells might be less immunogenic than WT skin cells. To test that, dLN T cells isolated from transplanted mice (day 4 post-transplant) were cultured with irradiated skin cells derived from WT or Batf3^−/−^ allografts, and IFN-γ production was analyzed by ELISPOT. We isolated skin stimulator cells from 24 h transplanted allografts when most of donor migratory cells were gone^19,20^. When we re-stimulated the WT or Batf3^−/−^-primed T cells with WT skin graft cells, we found less IFN-γ production in mice that received Batf3^−/−^ skins compared to WT (Fig. 2i). Furthermore, when we cultured WT-primed T cells with WT or Batf3^−/−^ skin allograft cells, we found that Batf3^−/−^ skin showed inferior allostimulatory capacity (Fig. 2i). T cells primed with Batf3^−/−^ allografts and re-stimulated with Batf3^−/−^ skins presented the lowest production of IFN-γ, indicating that donor CD103^+^ DCs are required in both priming and recall of alloreactive T cells. In summary, the lack of CD103^+^ DCs in the donor skin leads to less priming of alloreactive T cells in the dLN; and when these T cells migrate to the skin, they will also encounter less donor MHC II, which may weaken the rejection process. Overall, Batf3-deficient skins have less allo-MHC II skin tissues leading to reduced priming of allo-T cells on dLN and prolongation of allograft survival in the absence of systemic immunosuppressive treatment.

### DnaK treatment targets CD103^+^ DCs and impairs alloimmunity

**Fig. 3:**
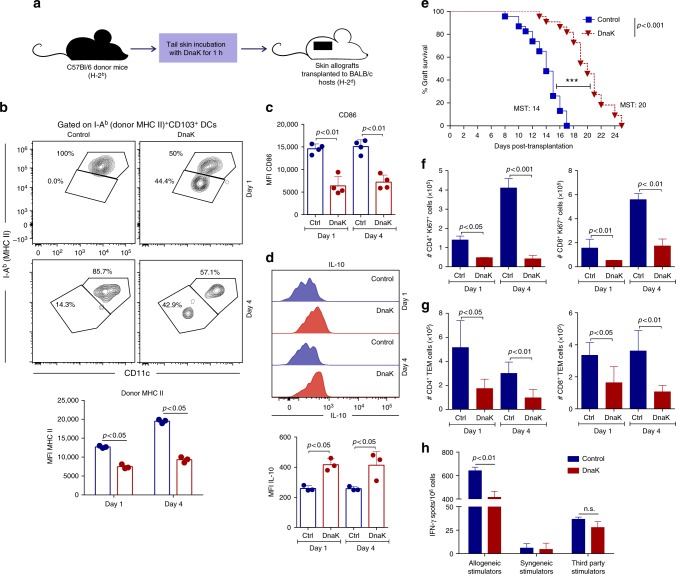
In situ skin treatment with DnaK prior to the transplant decreases donor MHC II expression in cross-dressed host DCs and improves allograft survival. Skin allografts from B6 mice (H-2K^b^/I-A^b^) were immersed in PBS solution with or without DnaK for 1 h, followed by transplantation into fully MHC mismatched BALB/c hosts (H-2K^d^/I-A^d^). **a** Schematic view of the experimental design. **b** MHC II, **c** CD86 and **d** IL-10 expression in dLN cross-dressed host CD103^+^ DCs from mice that received DnaK-treated allografts or controls. Bar graphs represent the mean ± S.D. Statistics using *t* test. **e** Percent of graft survival after DnaK treatment (*n* = 23/group). Mean survival time (MST). Statistic by long-rank test. Results are pooled from three experiments. **f** Numbers of CD4^+^Ki67^+^ and CD8^+^Ki67^+^, and **g** CD4^+^ and CD8^+^ TEM cells from allograft dLNs harvested at day 1 and 4 post-transplant. Statistics using ANOVA with Tukey post-test (*n* = 3 mice per group). Bar graphs represent the mean ± S.D. **h** T cells from allograft dLN were magnetically isolated and cultured with allogeneic (B6), syngeneic (BALB/c) or third party (C3H, H-2K^k^/I-A^k^) irradiated splenocytes for 48 h in vitro in mouse IFN-γ-coated plates. IFN-γ production by T cells was measured by ELISPOT. Numbers of spots per million T cells ± S.D. in triplicates and pooled from three mice. Statistics using *t* test. Representative results of at least two independent experiments

Next, we explored if the modulation of donor-derived MHC molecules could be a potential way to attenuate alloimmunity. A potential candidate was DnaK from *M. tuberculosis*, since it had previously shown to be a potent anti-inflammatory molecule that modulates MHC II molecule expression on DCs^11,13^. Therefore, we performed a fully MHC-mismatched skin transplant, with an in situ treatment of donor skin prior to the transplant. We immersed B6 (H-2K^b^/I-A^b^) donor tissues in a solution containing DnaK and transplanted the skins into BALB/c (H-2K^d^/I-A^d^) hosts (Fig. 3a). DnaK treatment decreased the expression of donor MHC II (I-A^b^, Fig. 3b) and CD86 (Fig. 3c) of cross-dressed host CD103^+^ DCs in dLN, both on day 1 and 4 post-transplant. In addition, CD103^+^ DCs within dLNs of DnaK-treated allografts produced more IL-10 than non-treated controls (Fig. 3d). Migration of donor DCs was not affected by DnaK treatment (Supplementary Fig. 5).

Consistent with our previous observation^21^, DnaK-treated donor skins presented significantly prolonged survival without any additional treatment (Fig. 3e). DnaK-treated grafts had a median survival time (MST) of 20 days compared to 14 days for the control group (*n* = 23 per group; *p* < 0.0001). We obtained similar results in a different MHC mismatch context, in which donor C3H skin grafts (H-2K^k^/I-A^k^) were incubated with DnaK and transplanted to BALB/c (H-2K^d^/I-A^d^) hosts (Supplementary Fig. 6). This prolongation of graft survival with DnaK is significant for a stringent model as skin transplantation in which interventions such as anti-CD8 depletion^22^ and immunosuppression with CTLA-4-Ig^23^ were incapable of prolonging graft survival. DnaK treatment of donor skins also significantly decreased the absolute numbers of proliferating CD4^+^ and CD8^+^ T cells (Fig. 3f), and CD4^+^ and CD8^+^ TEM cells (Fig. 3g).

We next isolated dLN T cells isolated from DnaK-treated animals or controls, exposed them to irradiated allogeneic (B6) splenocytes and evaluated IFN-γ production by ELISPOT. Decreased numbers of IFN-γ–producing T cells were detected in animals that received DnaK-treated allografts (Fig. 3h). We observed no changes in response to third-party stimulators in the DnaK-treated group compared with controls (Fig. 3h), suggesting that DnaK reduced the priming of donor-specific T cells. DnaK did not affect the indirect allorecognition pathway, because B6 DCs from hosts that received DnaK-treated BALB/c skin transplants could present the Eα_52–68_ peptide (BALB/c-derived) in I-A^b^ context^24^ in similar way than controls (Supplementary Fig. 7). In sum, DnaK treatment of donor skins reduces the expression of donor MHC II on host cross-dressed CD103^+^ DCs, decreasing the priming of anti-donor T cells and improving graft survival with no additional therapy.

### DnaK induces March1-dependent MHC II surface downregulation

**Fig. 4:**
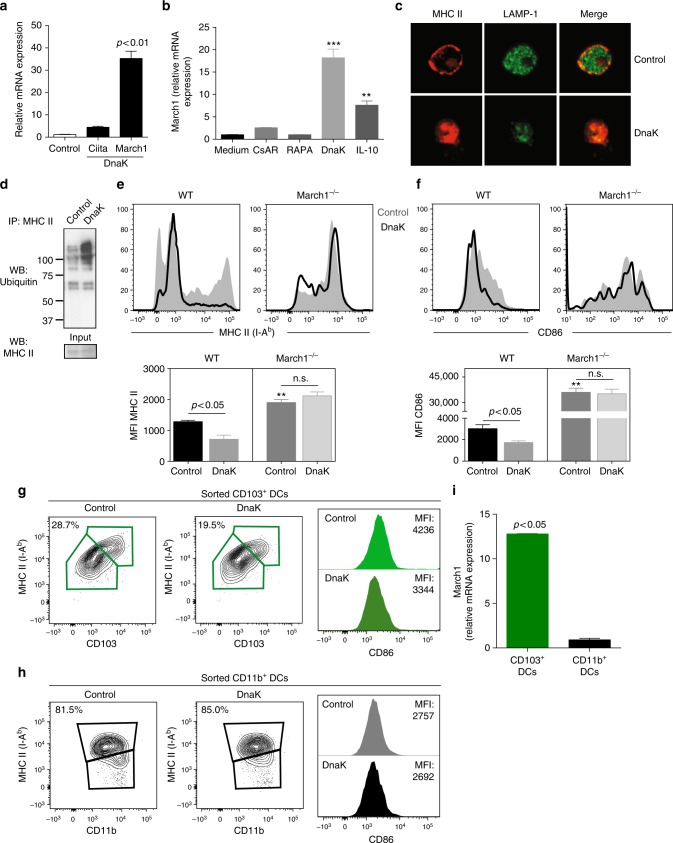
DnaK induces MHC II downregulation through induction of March1. **a** *March1* and *Ciita* mRNA levels in DCs isolated from mice lymph nodes (LN DCs) and treated with DnaK. **b** March1 mRNA expression in LN DCs treated with CsA, RAPA, DnaK, or recombinant murine IL-10 for 24 h. *Actb* was used as internal control. Relative expression compared to untreated cells. **c** Confocal microscopy of LN DCs treated with DnaK or medium for 24 h and stained for MHC II and lysosomal-associated membrane protein 1 (LAMP-1) using anti-MHC II (red, secondary) and anti-LAMP-1 antibodies (green). **d** MHC II proteins were immunoprecipitated from LN DCs treated with DnaK and analyzed for ubiquitination or total MHC II by Western Blot. **e** MHC II and **f** CD86 expression on WT or March1^−/−^ DCs treated with DnaK. **p < 0.01 when compared to WT control. **a**–**f** DCs were isolated with anti-CD11c-coated magnetic beads. Statistics using ANOVA with Tukey post-test. MHC II expression (contour plots) and CD86 MFI on skin-dLN flow sorted CD103^+^ (**g**) or CD11b^+^ DCs (**h**) after treatment with DnaK. **i** March1 mRNA expression in skin-dLN flow sorted CD103^+^ or CD11b^+^ DCs after treatment with DnaK. Statistics using *t* test. For all experiments bar graphs represent the mean ± S.D., and *n* = 3 mice per group. Representative of at least two independent experiments

Next, we sought to address the molecular mechanisms by which DnaK induces the downregulation of MHC II and CD86 in DCs. MHC II expression can be regulated by transcriptional and post-translational mechanisms. While transcription of MHC II is regulated by the MHC class II transactivator (CIITA), membrane-associated RING-CH 1 (March1) can ubiquitinate MHC II, leading to internalization and downregulation from the cell surface. DnaK is a strong inducer (~35-fold increase) of March1, but does not alter CIITA in murine LN DCs (Fig. 4a). We then compared DnaK ability to induce March1 with molecules that have been reported to modulate DC activity and inhibit allograft rejection, such as cyclosporin A (CsA)^25,26^ and rapamycin (RAPA)^27^. Because IL-10 is known to induce March1^16^, IL-10 was used as positive control. DnaK and recombinant murine IL-10 treatments, but not CsA or RAPA, led to a significant induction of March1 mRNA levels in murine DCs (Fig. 4b). To assess whether ubiquitination targets MHC II towards late lysosomal vesicles, we analyzed the localization of MHC and late endosomes/lysosomes marker lysosomal-associated membrane protein 1 (LAMP-1) in DCs treated with DnaK using confocal microscopy. In DnaK-treated DCs, MHC II was internalized and co-localized with LAMP-1 in intracellular vesicles, while untreated DCs showed MHC II on the cell surface and LAMP-1 distributed intracellularly with minimal overlap with MHC II (Fig. 4c). In addition, ubiquitination of MHC II was increased in DCs treated in vitro with DnaK when compared to untreated DCs (Fig. 4d). Since CD86 expression levels can also be regulated by March1^28^, we next assessed MHC II and CD86 expression by flow cytometry and found that DnaK was able to downregulate MHC II (Fig. 4e, f) and CD86 (Fig. 4g, h) expression in WT DCs, but not in March1^−/−^ cells, indicating that March1 is required for DnaK-induced downregulation of MHC II and CD86. DnaK treatment could decrease MHC II and CD86 levels only in the CD103^+^ DCs (Fig. 4g), but not in CD11b^+^ DCs (Fig. 4h) sorted from skin-dLNs. Also, March1 was induced only in CD103^+^, but not in CD11b^+^ DCs (Fig. 4i) upon DnaK stimulation, demonstrating its specific effect on this DC subset.

### March1 is induced via ERK/STAT3/IL-10 pathway

**Fig. 5:**
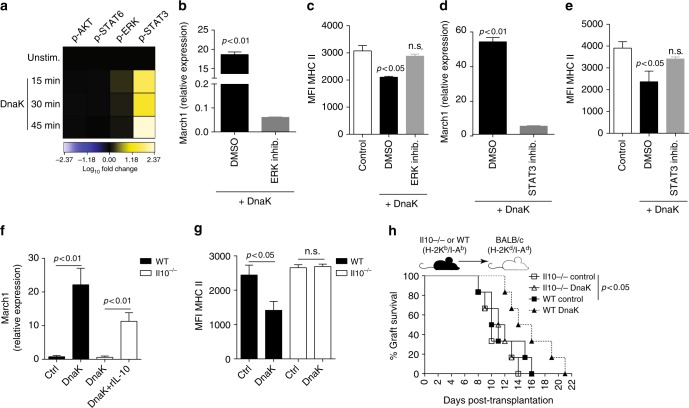
March1 induction by DnaK requires ERK, STAT3 and IL-10. **a** BioHeat map representing the fold change of p-Akt (pS473), p-STAT6 (pY641), p-ERK1/2 (pT202/pY204), and p-STAT3 (pY705) in the BMDCs treated with DnaK for 15, 30, and 45 min, compared to untreated ones. March1 mRNA levels and MHC II levels by flow cytometry on WT LN DCs treated with **b**, **c** ERK inhibitor (PD98059) or **d**, **e** STAT3 inhibitor (BP-1-102) and stimulated with DnaK. **f** March1 expression in WT or Il10^−/−^ LN-derived DCs treated with DnaK, medium or DnaK + recombinant murine IL-10. *Actb* was used as internal control for real-time PCR analysis. **g** MHC II levels on WT or Il10^−/−^ DnaK- or control-treated DCs. Statistics using ANOVA with Tukey post-test (*n* = 3 mice per group). Bar graphs represent the mean ± S.D. Representative of at least two independent experiments. **h** Skin allografts survival from WT or Il10^−/−^ (H-2K^b^/I-A^b^) mice treated with DnaK or control and transplanted into BALB/c (H-2K^d^/I-A^d^) recipients. Statistics by log-rank test (*n* = 6/group). Representative of three independent experiments

We further investigated the mechanisms underlying DnaK-mediated induction of March1 and downregulation of MHC II levels in DCs. We previously demonstrated that DnaK could modulate DC properties in a pathway involving extracellular signal-regulated kinase (ERK) and signal transducer and activator of transcription 3 (STAT3)^29^. To better characterize this signaling pathway, we treated bone marrow-derived dendritic cells (BMDCs) with DnaK for 15, 30, and 45 min and then analyzed the expression of p-Akt (pS473), p-STAT6 (pY641), p-ERK1/2 (pT202/pY204), and p-STAT3 (pY705) by flow cytometry. We did not observe any change in p-Akt (Fig. 5a, lane 1) and p-STAT6 (Fig. 5a, lane 2) upon DnaK treatment. However, DnaK increased p-ERK1/2 levels at 15 min post-stimulation (Fig. 5a, lane 3), and p-STAT3 at 45 min (Fig. 5a, lane 4). To test whether these two molecules were required for DnaK-induced March1 expression, we utilized MEK inhibitor—PD98059^30^ to inhibit ERK and a specific p-STAT3 inhibitor—BP-1-102^31^. March1 expression was completely inhibited in PD98059- (Fig. 5b) and BP-1-102-treated DCs (Fig. 5d). In addition, both molecular pathways were required for MHC II downregulation (Fig. 5c, e) in DnaK-treated DCs, suggesting ERK-STAT3 axis plays a role in upregulation of March1.

IL-10 is a major anti-inflammatory cytokine that downregulates MHC II expression^32^ through the induction of March1^16^. In addition, DnaK has been shown to increase IL-10 production by BMDCs and macrophages^11,33^, and its anti-inflammatory effects depend on this cytokine^34,35^. We tested whether induction of IL-10 by DnaK was the basis for March1 induction and MHC II downregulation. We treated DCs isolated from WT or Il10^−/−^ mice with DnaK in vitro and analyzed March1 expression. We observed that Il10^−/−^ DCs treated with DnaK did not undergo upregulation of March1 compared to WT DCs (Fig. 5f). The addition of recombinant murine IL-10 partially restored March1 expression (Fig. 5f). Furthermore, IL-10 expression in these cells was required for DnaK-induced downregulation of MHC II (Fig. 5g). These findings are consistent with the observed loss of DnaK-mediated graft protection in Il10^−/−^ skin grafts (H-2K^b^/I-A^b^) transplanted in BALB/c hosts (Fig. 5h). We concluded that March1 induction by DnaK requires the ERK-STAT3-IL-10 molecular pathway.

### DnaK impairs alloimmunity through March1

**Fig. 6:**
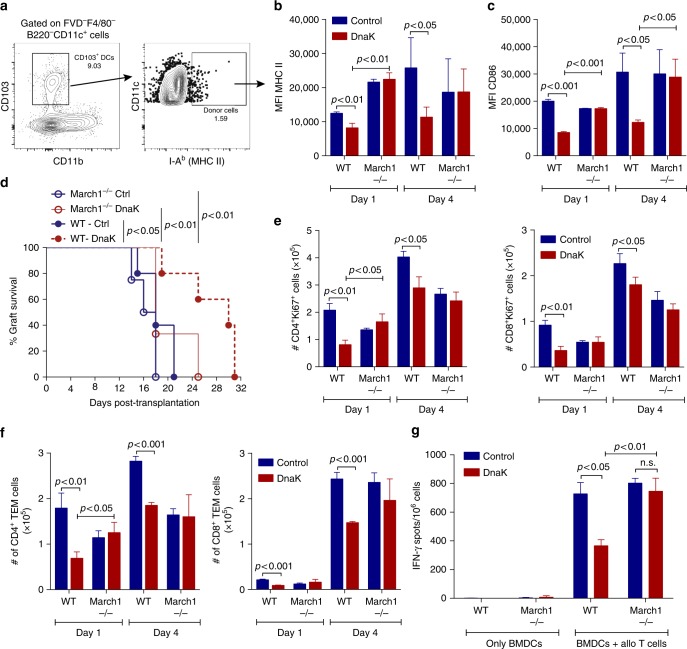
March1 is required for dampened alloreactivity induced by DnaK. Skin allografts from WT or March1^−/−^ mice (both H-2K^b^/I-A^b^) were treated with DnaK or control and transplanted into BALB/c (H-2K^d^/I-A^d^) recipients. **a** Gating strategy for tracking donor MHC II (I-A^b^) in cross-dressed host CD103^+^ DCs. **b** Donor MHC II (I-A^b^) and **c** CD86 levels in cross-dressed host CD103^+^ DCs graft dLN at day 1 and 4 post-transplant. **d** Percent of allograft survival. Statistics using log-rank test (*n* = 5/group). **e** Numbers of CD4^+^Ki67^+^ and CD8^+^Ki67^+^ T cells, and **f** CD4^+^ and CD8^+^ TEM cells from allograft dLNs harvested at day 1 and 4 post-transplant. Statistics using ANOVA with Tukey post-test. *n* = 3 mice per group. Bar graphs represent the mean ± S.D. **g** WT or March1^−/−^ BMDCs were treated with DnaK for 24 h, irradiated and cultured with allogeneic T cells for 24 h in mouse IFN-γ-coated plates. IFN-γ production by T cells was measured by ELISPOT. Numbers of spots per million T cells ± S.D. in triplicates. Statistics using ANOVA with Tukey post-test. Representative results of at least two independent experiments

Given the DnaK-induced March1 expression and MHC II downregulation in DCs in vitro, we evaluated the involvement of March1 in DnaK-mediated attenuation of alloimmune responses in vivo. We treated WT or March1^−/−^ skin grafts (H-2K^b^/I-A^b^) with DnaK, transplanted them in BALB/c hosts and analyzed I-A^b^-expressing CD103^+^ DCs, as shown in Fig. 6. In March1^−/−^ CD103^+^I-A^b+^ DCs from dLNs, the expression levels of MHC II or CD86 were unchanged with DnaK treatment (Fig. 6a–c), both on day 1 and 4 post-transplant, compared to WT cells. Importantly, the improvement in allograft survival induced by in situ treatment with DnaK was dependent on March1 expression by donor skins (Fig. 6d). In accordance, we observed a significant reduction in absolute numbers of proliferating (Ki67^+^) CD4^+^ and CD8^+^ T cells (Fig. 6e) in hosts that received DnaK-treated allografts. This reduction was completely abolished when donor skins were March1-deficient (Fig. 6e). Consistent with reduced allosensitization, mice that received DnaK-treated allografts showed a March1-dependent reduction in absolute numbers of CD8^+^ and CD4^+^ TEM cells (Fig. 6f). All T cells were from host origin (Supplementary Fig. 8). We next tested whether the decrease in the DCs allostimulatory capacity induced by DnaK was March1-dependent. We treated WT or March1^−/−^ BMDCs with DnaK, cultured them with allo-T cells and evaluated T cells IFN-γ production by ELISPOT. DnaK decreased the capacity of WT, but not March1^−/−^, BMDCs to induce IFN-γ production by allo-T cells (Fig. 6g). These results indicated that in situ DnaK treatment downregulates donor MHC II and CD86 surface levels through March1 both in vitro and in vivo. Also, March1 induction by DnaK was essential for decreased alloreactivity to skin transplants, modulation of T cell activation and improvement in allograft survival.

To assess the contribution of donor MHC II to graft rejection and T cell responses in our model, we isolated dLN T cells from animals that received fully-mismatched WT or MHC II^−/−^ skins grafts, cultured them with allogeneic (B6) stimulator (direct allorecognition), syngeneic (BALB/c) or third-party (C3H—H-2K^k^/I-A^k^) cells and analyzed IFN-γ production by ELISPOT. T cells from animals that received MHC II^−/−^ grafts produced less IFN-γ when cultured with allogeneic splenocytes (Supplementary Fig. 9a). The same did not happen when T cells were cultured with third-party stimulators. When incubated in vitro with MHC II^−/−^ allogeneic stimulators, we also observed less IFN-γ by T cells from BALB/c mice transplanted with WT B6 skins (Supplementary Fig. 9b). These data suggest that skin transplant is generating donor MHC II-specific T cell responses. Overall, DnaK reduces donor MHC II expression on CD103^+^ DCs via March1, impairing alloreactivity to skin allografts.

### DnaK decreases allo-stimulatory capacity of human DCs

**Fig. 7:**
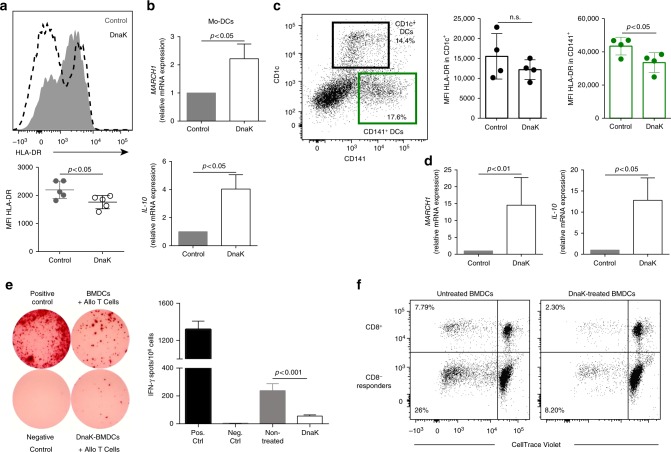
DnaK reduces HLA-DR surface expression, induces MARCH1 and modulates allo-stimulatory capacity of human dendritic cells. **a** Representative histogram (upper) and mean fluorescence intensity (MFI) quantification (lower) of HLA-DR expression in DnaK- or control-treated human monocyte-derived (Mo-DCs). Represented as mean ± SEM. *p < 0.05 when compared by Mann–Whitney. **b** Relative *MARCH1* (upper) and *IL-10* (lower) mRNA expression by DnaK-treated human Mo-DCs compared to non-treated cells. **c** Representative dot plot of human skin dermal DCs and HLA-DR MFI in CD141^+^ and CD1c^+^ DCs treated with DnaK. **d** Relative *MARCH1* (left) and *IL-10* (right) mRNA expression by DnaK-treated human skin cells. For RT-PCR, *GAPDH* was used as internal control. Represented as mean ± SEM. Statistics using Mann–Whitney test. **a**–**d** *n* = 4–5 subjects. DnaK-treated or control BMDCs were assessed for their capacity to stimulate (**e**) IFN-γ and **f** proliferation of allogeneic PBMCs. **e**, **f** *n* = 3 subjects. Bar graphs represent the mean ± SD. Statistics using ANOVA with Tukey post-test

We next asked whether the modulation of MHC II expression via DnaK-induced March1 would also occur in human cells. Thus, we investigated DnaK-mediated MHC II downregulation using human peripheral monocyte-derived dendritic cells (Mo-DCs), as well as human skin-derived DCs. First, Mo-DCs were differentiated from five healthy volunteers, and were cultured in the presence of DnaK for 24 h. DnaK-treated Mo-DCs had lower HLA-DR (MHC II) surface expression levels (Fig. 7a), higher MARCH1 and IL-10 mRNA levels (Fig. 7b). Similarly, when treated with DnaK for 24 h, human skin-derived primary DCs showed lower HLA-DR surface expression levels only in CD1c^−^CD141^+^ population, which corresponds to mouse CD103^+^ DCs, but not in CD1c^+^CD141^−^ DCs (Fig. 7c; Supplementary Fig. 10). MARCH1 and IL-10 mRNA levels were also increased in skin cells (Fig. 7d). Furthermore, to functionally assess the modulation of allo-stimulatory capacity of DnaK-treated DCs, human BMDCs were co-cultured with third party peripheral blood mononuclear cells (PBMCs) and IFN-γ production was measured by ELISPOT. Human BMDCs pre-treated with DnaK-induced significantly less allo-T cell IFN-γ production compared to those cultured with non-treated BMDCs (Fig. 7e). In accordance, in mixed lymphocyte reaction, DnaK-pre-treated BMDCs provoked less proliferation of CD4^+^ and CD8^+^ T cells compared with those co-cultured with non-treated BMDCs (Fig. 7f). Overall, our findings suggest that DnaK-mediated downregulation of MHC II surface expression in DCs can inhibit human alloimmune responses in vitro.

## Discussion

The contribution of each skin DC subset to initiate alloimmune responses is still being elucidated. While donor LCs are not required for the complete rejection of MHC-mismatched skin allografts^36^, Celli et al.^20^ demonstrated by intravital microscopy that dermal DCs rapidly migrated from the skin to host dLNs^20^. However, the specific dermal DC subsets were not distinguished in that study. We found that donor-derived CD103^+^ was the major DC subset carrying donor MHC to dLNs. The importance of this subset is reinforced by the fact that skins lacking the CD103^+^ DCs (Batf3^−/−^) had better graft survival with reduced donor MHC II expression in the dLN and allografts, and less primed T cells. Recently, donor CD103^+^ DCs were shown to have a major role in amplifying the pathology of graft-versus-host disease^37^. *Batf3*-dependent DCs were also proposed to have a role in rejection of minor mismatched grafts^38^. Our data highlights that donor CD103^+^ DCs is the dominant cell source transferring donor MHC II molecules to dLN, either by direct migration in early time-points or by cross-dressing of host DCs. However, the precise contribution of each of these scenarios in the priming of allo-T cells needs to be further explored. This is the first report to demonstrate the key role of donor *Batf3*-dependent DCs in initiating acute rejection of a fully MHC-mismatched graft model, identifying this subset of DCs as the major source of donor MHC II, and a potential target to modulate the alloimmune response.

While direct and indirect allorecognition have been traditionally considered the primary mechanisms of DC activation of allo-specific T cells, the importance of semi-direct allorecognition is being increasingly recognized^4,39,40^. The shuttle of donor MHC antigens through extracellular vesicles has been shown to dominate early allorecognition compared to direct contact of donor DC with host T cells, since only few donor DCs were identified in dLN at 24 h after transplantation^4,40^. The extracellular vesicles are uptaken and incorporated onto host DC cell membranes (cross-dressing), and these host DCs then activate alloreactive T cells^4,40^. The source of donor-derived vesicles still need to be investigated, since most of I-A^b+^ events are CD11c^−^ from 3 to 12 h post-transplant (Fig. 1c). Nonetheless, we could detect in very early time-points (3–12 h) after transplant that a high percentage (~40–60%) of donor MHC II^+^ cells did not express host MHC, and therefore were donor DCs. This discrepancy between studies seems to be primarily related to the timing of analyses (3–12 h vs. 24 h), indicating that in our model donor DCs and donor-derived vesicles are reaching the dLN and cross-dressing host DCs at later time-points (24 h)^19^. From these host cross-dressed DCs, most of cells carrying I-A^b^ were also CD103^+^ DCs in very early time-points. It is possible that CD103 molecules expressed on membrane of donor cells are transferred and incorporated on membrane of DCs from a different origin, like CD103^−^CD11b^−^ DCs^41,42^. Another possibility is that the origin of the extracellular vesicles would dictate the cell type in which these vesicles will be incorporated. Also, CD103^+^ DCs might produce more extracellular vesicles and have a superior capacity to cross-dress host DCs compared to other subsets, characterizing this subset as the main provider of donor MHC II during skin transplantation. However, these possibilities need to be further tested. Overall, direct and semi-direct allorecognition are important early after transplant with the latter becoming dominant as migratory CD103^+^ donor DCs are killed by host cells^19^. Identification and targeting of specific graft-derived cell subsets to decrease the load of donor MHC may allow for effective immune modulation to be achieved with lower immunosuppressant dosage, thereby decreasing side-effects and toxicity.

The expression of donor MHC II is critical for induction of allograft rejection. Donor, but not host, MHC II expression is required for CD4^+^ T cell-mediated rejection in a mouse model of cardiac transplants^43^. The absence of surface MHC II in donor cells results in significant prolongation of primary cardiac allograft survival^44^. Nevertheless, complete eradication of donor MHC II in order to induce transplant tolerance in vivo has not yet been considered a possibility^45^. Targeting costimulatory ligands B7 and CD40 has shown promise in clinical trials^46^, though deleterious effects in regulatory T cells (Tregs) and lack of effect in memory T cells are significant limitations^47,48^. Low MHC II expression not only constitutes reduction of signal 1 into T cells; it also actively promotes tolerogenic responses^49,50^. Regulatory DCs (DCregs) are characterized by the low constitutive expression of surface MHC molecules and low net expression of costimulatory molecules^49^. Such cells are functionally more resistant to danger signals received through TLRs, promoting apoptosis of effector T cells and generating Tregs. In transplantation, different approaches have been attempted to harness the therapeutic potential of such cells, including the administration of donor DCregs or recipients’ DCregs pulsed with donor antigen prior to transplantation^51,52^. A major risk with these approaches is the sensitization of the recipient to donor antigens, leading to the production of alloantibodies and antibody-mediated rejection, both clearly documented in prior studies^53,54^. Our study offers an alternative approach that can circumvent such risks by manipulating donor MHC II levels, reducing early innate immune activation critical to trigger T cells in the setting of ischemia-reperfusion injury of allografts. Whether this approach would induce a tolerant state to donor antigens in combination with other immunosuppressive drugs requires further investigation.

Optimal T cell activation requires three signals: (i) recognition of T cell receptor by a peptide:MHC complex; (ii) co-stimulatory receptors, mainly engagement of CD28 on the T cell by B7 molecules on DCs and (iii) production of cytokines by DCs which will shape the type of T cell response^55^. Immune checkpoint modulators are emerging as powerful therapeutic tools for use in tumor immunology, but also in autoimmunity, transplantation, and asthma. In transplantation, most of such therapies focus on co-stimulatory (signal 2) blockade. The effect of DnaK on CD103^+^ DCs demonstrated in this study highlights a therapeutic potential for mitigating all three signal steps. The use of other immunosuppressive drugs such as CTLA-4-Ig^23^ and anti-CD8^22^ is not enough to improve skin graft survival like DnaK was capable of. Since we had immersed donor skin tissues in a solution containing DnaK (Fig. 3a), it is possible that part of the effect observed up to 4 days after transplantation (after donor DCs were gone from dLN—Fig. 3b–g) could be related to an effect of DnaK directly in host cross-dressed CD103^+^ DCs. However, our findings that DnaK could not prolong allograft survival when skin grafts were IL-10- (Fig. 5) or March1-deficient (Fig. 6) indicates that DnaK’s effect is primarily occurring on donor DCs. The sole administration of DnaK to the donor organ has a putative advantage in minimizing any potential side effect in the recipient. The complexity of signals required for rejection modulation in a long-term is a clinical challenge that will certainly involve a combination of different and complementary drug targets. Therefore, the identification of other molecules capable of inducing March1 in APCs could thus constitute a promising line of investigation in the modulation of inflammatory disorders.

Consistent with previous studies, we demonstrated ERK activity to modulate IL-10 production^56,57^. This is the first report to link ERK and STAT3 in the regulation of March1 expression, advancing the known scope for ERK-STAT3 signaling in immune regulation. Indeed, this study identifies a novel mechanism of how STAT3 activation may dampen an immune response via March1-mediated downregulation of MHC II. The molecular contexts which govern the immunological outcome of STAT3 signaling are beyond the scope of this study. However, as shown here, STAT3 may be targeted by agents such as DnaK to favor a desired immunological state for therapeutic purpose. DnaK also induced March1 in an IL-10-dependent manner. IL-10 is a major anti-inflammatory cytokine and is required for early acceptance of skin allografts^58^. In human monocyte-derived macrophages, STAT3 can bind to the IL-10 promoter and induce IL-10 production^59^. In addition, IL-10 exerts its anti-inflammatory role in a STAT3-dependent manner^60^. In our model, DnaK triggers IL-10 signaling, activates STAT3 and induces the expression of March1. These results underline the intricacy of pathway interactions necessary for the induction of March1. Although Mittal et al.^61^ has shown that IL-10 modulates MHC II and CD86 independently on March1 induction in DCs, other studies have demonstrated that the two are connected in control of MHC II expression in DCs^17,28,62^. Thus, the simplest explanation for our findings is that DnaK can activate other signaling pathways concomitantly with the IL-10 axis. One evidence supporting this line of thought is that when we treat DCs with dexamethasone (Fig. 4a), a known IL-10 inducer, this is not enough to upregulate March1 expression in DCs.

We found that DnaK can induce MARCH1 and downregulate MHC II levels in human monocyte-, bone-marrow-derived and skin DCs. Given the importance of these cell types and MHC II to alloreactive responses, our findings are relevant for the already evident critical role of innate immune activation in precipitating rejection and reducing graft survival^63–65^. Prolonged ischemic time of the graft correlates with higher rejection rates in deceased donor organ transplants^63,64^, and is associated with higher MHC expression^65^, demonstrating the impact of enhanced innate components due to severe ischemia on adaptive immunity. Therefore, the development of therapies targeting the donor organ without systemic toxicity are greatly warranted. DnaK treatment of skin allograft could thus pave the way for new strategies to modulate donor MHC II expression prior to transplant and locally reduce early alloimmune responses. Further studies, in combination with other immunosuppressive drugs, are necessary to determine the benefits of this strategy in transplant outcomes.

## Methods

### Mice

BALB/c (H-2K^d^/I-A^d^) and C57Bl/6 (B6, H-2K^b^/I-A^b^) mice were purchased from Fundação Estadual de Produção e Pesquisa em Saúde (FEPPS—Rio Grande do Sul, Brazil) or from The Jackson Laboratory. B6 CD45.1 (B6.SJL-Ptprca Pepcb/BoyJ) and MHC II^−/−^ (B6.129S2-H2dlAb1-Ea/J) mice were purchased from The Jackson Laboratory. C3H/HeJ (C3H, H-2K^k^/I-A^k^) mice were provided by Dr. Niels Câmara (University of São Paulo, São Paulo, Brazil) or purchased from The Jackson Laboratory. March1^−/−^ (H-2K^b^/I-A^b^) mice^14^ were provided by Dr. Jeoung-Sook Shin (University of California, San Francisco, USA). Batf3^−/−^ mice were both generated and maintained as breeding colonies in Harvard Medical School facility. Il10^−/−^ (H-2K^b^/I-A^b^) mice were provided by Dr. Ana M. C. Faria (Federal University of Minas Gerais, Belo Horizonte, Brazil). C57BL/6-GFP (H-2K^b^/I-A^b^) mice were provided by Dr. Gustavo B. Menezes (Federal University of Minas Gerais, Belo Horizonte, Brazil). All mice used in the experiments were females between six to 10 weeks old. Animals were bred and housed in individual and standard mini-isolators under specific pathogen-free conditions at the School of Biosciences—Pontifícia Universidade Católica do Rio Grande do Sul (PUCRS) or Harvard Medical School facility. All animals were housed in accordance with the Institutional Animal Care and Use Committee (IACUC) and National Institutes of Health (NIH) Animal Care guidelines.

### Protein purification and endotoxins removal

The *M. tuberculosis* DnaK gene (Gene ID: 885946) was synthesized, subcloned and cloned into the pET23a(+) vector at the NdeI and BamHI sites by GenScript (USA). BL21(DE3) *Escherichia coli* cells were transformed, and the expressed protein was purified according to methods described previously^66^. To remove lipopolysaccharide (LPS), Triton X-114 was used according to the method described in Aida, et al^67^. Residual Triton X-114 (Sigma) was removed by overnight incubation with Bio-Beads (Bio-Rad) at 4 °C. Protein concentration was determined using a Qubit Protein Assay Kit (Invitrogen), which was read on a Qubit® Fluorometer (Invitrogen). Protein integrity was analyzed by Western Blot using anti-Hsp70 antibody (clone C92F3A-5—StressMarq; Supplementary Fig. 11a). Endotoxin levels were measured during all purification steps (Supplementary Fig. 11b) using a chromogenic limulus amebocyte lysate (LAL) endotoxin assay kit (GenScript). Only samples with endotoxin levels below 0.1 EU/mL were used. To check protein purity, we took advantage of the fact that the splenic DCs upregulate CD86 levels after 6 h of intravenous injection of LPS^68^. We intravenously injected B6 mice with either 25 µg of LPS, 30 µg of DnaK, 30 µg of Triton X-114-treated DnaK, or control (phosphate-buffered saline [PBS] treated with Bio-Beads). Mice were sacrificed after 6 h and analyzed for CD86 expression in splenic DCs (CD11c^+^) by flow cytometry^35^. LPS and non-treated DnaK increased the expression of CD86 in splenic DCs. In contrast, mice injected with DnaK or PBS treated with Triton X-114 showed no upregulation of CD86 in splenic DCs (Supplementary Fig. 11b). DnaK has adenosine triphosphatase (ATPase) activity, so we tested our purified protein for its ability to hydrolyze adenosine triphosphate (ATP). Functional DnaK (5 µg) and nonfunctional DnaK (5 µg) were incubated at 37 °C in a reaction medium containing 2 mM calcium chloride, 120 mM sodium chloride (NaCl), 5 mM potassium chloride, 10 mM glucose, 20 mM hydroxyethyl piperazineethanesulfonic acid (HEPES) with 100 µM ATP at pH 7.4 for 0, 10, 20, 60, and 120 min. After the incubation, the reaction medium was transferred to ice and centrifuged at 10,000 rpm for 30 min at 4 °C. Aliquots of 40 μL supernatant were injected onto a C18 reverse-phase high performance liquid chromatography (HPLC) column using a mobile phase containing 60 mM potassium dihydrogen phosphate (KH_2_PO_4_, Sigma-Aldrich) and 5 mM tetrabutylammonium chloride (Sigma-Aldrich), pH 6.0, in 30% methanol. All peaks were identified by retention time compared to an ATP standard curve. The control for non-enzymatic hydrolysis of nucleotides was performed by measuring the peaks present in the same reaction medium incubated without proteins. The results are expressed as total amount of the ATP (µM) at the respective incubation time (Supplementary Fig. 11c).

### Skin transplant and in situ treatment

We performed a fully MHC-mismatched murine skin allograft model^69^. B6 WT, March1^−/−^, MHC II^−/−^ or Il10^−/−^ mice (all H-2K^b^/I-A^b^), or C3H (H-2K^k^/I-A^k^) were used as donors. Briefly, 1 cm^2^ sections of tail skin were removed and immersed in PBS or PBS containing 60 µg/mL of purified DnaK for 60 min at 4 °C. BALB/c (H-2^d^/I-A^d^) recipients were anesthetized, and fur was shaved off the dorsal trunk. At the shaved area, 1 cm^2^ of skin was removed in each recipient mouse, and donor tail skin fragment was sutured to the exposed tissue of each recipient. Animals were kept in individual cages and the state of graft acceptance was photographed and recorded daily. Graft rejection was confirmed by the observation of cyanosis, erythema, erosion, and loss of skin graft.

### PBMCs isolation from healthy volunteers’ blood

Peripheral blood samples were obtained from healthy subjects and peripheral blood mononuclear cells (PBMCs) were then isolated by density gradient centrifugation using Ficoll-Paque solution (GE Healthcare). Cells were washed in PBS and plated for dendritic cells differentiation.

### Murine dendritic cell cultures

CD11c^+^ cells were isolated from LNs of B6 WT, March1^−/−^ and Il10^−/−^ mice. LNs were disrupted against a nylon screen and treated with Collagenase D (Roche) for 30 min at 37 °C. The resultant single cell suspensions were labeled with anti-CD11c (N418) magnetic beads (Miltenyi). After washing, CD11c^+^ cells were purified by positive selection using MACS separation columns (Miltenyi). Purity (≥85%) of selected cells (CD11c^high^MHC II^high^ cells) was controlled by flow cytometry analysis before and after the cultures. Cells were cultured in 96-well plates in AIM-V serum-free medium (Gibco). DCs were incubated media (control) or media containing 30 µg/mL of DnaK for 24 h and analyzed by flow cytometry or total RNA was extracted. The supernatant was collected and used for cytokine analysis.

DCs were grown from murine bone marrow (BMDCs) in the presence of granulocyte-macrophage colony-stimulating factor (GM-CSF) and IL-4 (both from Peprotech). Cells were cultured in 24-well plates in AIM-V serum-free medium (Gibco). Non-adherent cells (DCs) were separated from adherent cells after six days in culture. BMDCs were incubated for 24 h with media alone (control), media containing either 30 µg/mL of DnaK, 10 µg/mL of peptidoglycan (Sigma) or 30 µg/mL of DnaK and 20 ng/mL of recombinant murine IL-10 (R&D System); they were analyzed by FACS or total RNA was extracted. Supernatants were collected and used for cytokine analysis.

Flow-sorted DC subsets were cultured in 96-well plates with AIM-V serum-free medium (Gibco) and treated with 30 µg/mL of DnaK for 24 h. Cells were then analyzed by flow cytometry or total RNA extraction. Purity (≥90%) of sorted cells was controlled by flow cytometry analysis before and after the cultures.

### Real time polymerase chain reaction

Total RNA was isolated from murine or human DC cultures using RNA Easy Kit (Qiagen). The concentration of the purified total RNA samples was measured using a Qubit RNA Assay Kit (Invitrogen) and read in a Qubit Fluorometer (Invitrogen). RNA 50 ng was reverse transcribed with 100 U of Sensiscript (Qiagen). cDNA concentrations were measured using the Qubit dsDNA HS Assay Kit (Invitrogen) and then read in a Qubit Fluorometer (Invitrogen). In a final volume of 10 µL, 8 ng of cDNA was amplified using the following Taqman Gene Expression Assays (ThermoFisher Scientific): Il10 (Mm00439614_m1), March1 (Mm00613524_m1), Actb (4352933E), MARCH1 (Hs00215631_m1), IL10 (Hs00961622_m1) ACTB (Hs01060665_g1) and GAPDH (Hs03929097_g1). Quantitative real-time polymerase chain reaction (PCR) was performed with a StepOne Real-Time PCR System (Applied Biosystems). The relative mRNA levels were calculated using the comparative C_t_ method, using the house keeping genes β-actin and GAPDH as an internal control. Non-treated DCs served as a reference for treated DCs.

### Immunofluorescence and microscopy

Isolated DCs were labeled or incubated with DnaK at 37 °C for 24 h, then fixed with 4% paraformaldehyde and permeabilized using 0.1% Triton-X 100 (for visualizing intracellular proteins) or not (for surface expression or binding) using 0.1% Triton X 100. Cells were stained with primary antibodies (anti-I-A/I-E [MHC II], clone M5/114, eBioscience; anti-LAMP-1, clone 1D4B, Abcam) and then washed three times with 1 × PBS and stained again with fluorophore-conjugated secondary antibodies. Fluorophores were visualized using the following filter sets: 488 nm excitation and band pass 505–530 nm emission filter for Alexa 488; 543 nm excitation and band pass 560–615 nm for Cy3/Alexa 564 nm; and 633 nm excitation, in Zeiss Confocal Microscopy.

### Major histocompatibility complex II immunoprecipitation

LN DCs were treated with or without DnaK. Cells were then washed with ice-cold Dulbecco’s PBS and lysed in NP-40 lysis buffer (containing 1% NP-40, 150 mM NaCl, 1 mM ethylenediaminetetraacetic acid (EDTA), 1 mM phenylmethylsulfonyl fluoride (PMSF), 1× HALT protease and phosphatase inhibitor cocktail (Thermo Scientific)). For immunoprecipitation (IP), 1 mg of cell extract was incubated with 5 µg of anti-I-A/I-E (MHC II—clone M5/114, eBioscience) for 2 h at 4 °C followed by incubation with 20 µL of protein A (50% slurry, GE Healthcare) plus sepharose beads for either 2 h at room temperature or overnight at 4 °C. The beads were then washed with NP-40 lysis buffer and complexes were eluted by boiling in Laemmli sample buffer. For Western Blotting analysis, precipitated protein was resolved by 4–15% gradient sodium dodecyl sulfate polyacrylamide gel electrophoresis (SDS-PAGE) and transferred to polyvinylidene fluoride (PVDF) membranes. Membranes were immunoblotted with primary antibodies (anti-ubiquitin mouse, clone P4D1, Cell Signaling) and later secondary antibodies that were horseradish peroxidase (HRP)-conjugated. The membrane reactions were visualized by Perkin Elmer-enhanced chemiluminescence reagents. For control input, 15–30 μg of protein were resolved by 4–15% gradient SDS-PAGE and transferred to PVDF membranes. Membranes were immunoblotted with primary antibodies and later secondary antibodies that were HRP-conjugated. The membrane reactions were visualized by Perkin Elmer-enhanced chemiluminescence reagents.

### Flow cytometry

The following antibodies were used for murine cells: CD4 (GK1.5), CD8 (53-6.7), Ki67 (B56), CD44 (IM7), CD62L (MEL-14), IFN-γ (XMG1.2), I-A^b^ (AF6-120.1), I-A^d^ (39-10-8), CD11c (HL3), CD45R/B220 (RA3-6B2), CD11b (M1/70), CD103 (M290), CCR7 (CD197—4B12), CD86 (GL1), CD19 (1D3), IL-10 (JES5-16E3), CD45.1 (A20), CD45.2 (104), p-Akt (pS473; M89-61), p-STAT6 (pY641; 18/P-Stat6), p-ERK1/2 (pT202/pY204; 20 A), and p-STAT3 (pY705; 4/P-STAT3) from BD Biosciences; CD207 (Langerin—eBioRMUL.2) and Y-Ae (eBioY-Ae) from eBioscience; H-2K^d^ (SF1-1.1), F4/80 (BM8), CD19 (6D5), Ly-6G (1A8), CD3 (145-2C11) and CD11c (N418) from Biolegend. For human cells, we used CD14 (M5E2), CD83 (HB15e), CD86 (2331/FUN-1), HLA-DR (TU36), CD3 (UCHT1) from BD Biosciences; CD45 (HI30), HLA-DR (L243), CD141 (M80), CD1c (L161), CD11c (3.9) from Biolegend. Cell suspensions were Fc blocked for 20 min on ice, and then surface markers were stained by incubation for 30 min with antibodies in 2% fetal bovine serum (FBS) in phosphate-buffered saline (PBS) on ice. Staining of Ki67, IL-10 and IFN-γ was performed by using the Fixation/Permeabilization Kit (eBioscience). For IFN-γ detection, cell suspensions were pre-incubated for 6 h with 50 ng/mL of phorbol 12-myristate 13-acetate (PMA), 500 ng/mL ionomycin, and GolgiStop (BD Biosciences) in 10% FBS RPMI before Fc blocking, followed by surface staining, permeabilization, and intracellular staining of IFN-γ. Cells were analyzed using FACSCantoII (BD Biosciences) and BD FACSDiva software (BD Biosciences). Flow sorting was performed on a FACS Aria II (BD Biosciences). Doublets were excluded based on forward scatter and side scatter characteristics (FSC/SSC) and dead cells by staining with Fixable Viability Dye eFluor 780 (eBioscience) or Zombie NIR Fixable Viability Kit (Biolegend). Data obtained were analyzed using Flowjo software (version X, Tree Star).

### Analysis of phosphorylated molecules

For signaling experiments, BMDCs were treated with 30 μM of selective MEK inhibitor PD98059 (Cayman Chemical) for 30 min, or 1 µM of p-STAT3 inhibitor BP-1-102 (Selleck Chem) for 24 h or DMSO prior DnaK stimulation. Cells were stimulated with 30 µg/mL of DnaK for 15, 30, or 45 min. Cells were fixed with Cytofix Buffer (BD Biosciences) for 10 min at 37 °C and permeabilized with Phosflow Perm Buffer III (BD Biosciences) for 30 min on ice. Then, cells were stained for p-Akt, p-STAT6, p-ERK, and p-STAT3. Cells were analyzed using FACSCantoII (BD Biosciences) and BD FACSDiva software (BD Biosciences). Data obtained were analyzed with Flowjo software (version X, Tree Star). BioHeat maps were generated with the web^−^based software Cytobank (www.cytobank.org).

### Generation of human dendritic cells

Monocytes were isolated from PBMCS by adherence to plastic at 37 °C, 5% CO_2_ for 2 h. Adherent cells were cultured in in 24-well plates in AIM-V serum-free medium (Gibco) supplemented with human GM-CSF and IL-4 (both at 50 ng/ml from Peprotech). After 5 days, human TNF-α (500 UI/ml, from Peprotech) was added for Mo-DCs maturation. On day 7, Mo-DCs were treated with 30 µg/mL of DnaK for 24 h. After that, cells harvested and analyzed by FACS or total RNA was extracted.

Bone marrow cells from healthy volunteers were obtained via BWH Tissue Bank and red blood cells were hemolyzed using ACK lysing buffer (Lonza). Single cell suspension was prepared in serum-free AIM-V medium (Gibco) at 1 × 10^6^ cells/ml and cultured for 5 days at 37 °C 5% CO_2_, in the presence of 20 ng/ml human IL-4 and 20 ng/ml human GM-CSF (Biolegend). IL-4 and GM-CSF were supplemented again on day 3 of culture. DnaK was added to the media at 30 µg/ml on the 6th day of culture and the cells were incubated for another 24 h before used for mixed lymphocyte reaction.

### Isolation and quantification of skin immune cells

Immune cells from human skin were isolated, as described previously^70^. Briefly, skin tissues were minced into small pieces in 10% FCS-supplemented RPMI, followed by incubation in Collagenase D (Sigma, 0.2%) and DNAse (Invitrogen, 30 Kunitz Units/mL) at 37 °C for 2 h with shaking. After passing through 70 µm cell-strainer, cells were washed and recovered in RPMI media (Lonza) supplemented with 20% FCS, 100 mM l-glutamine and penicillin/streptomycin at 37 °C. Total skin cell numbers were calculated using fluorescent AccuCheck Counting Beads (Invitrogen). Beads were added to each sample after the final wash step, and cells were analyzed on a FACSCantoII flow cytometer (BD Biosciences). Data obtained were analyzed with Flowjo software (version X, Tree Star).

### IFN-γ ELISPOT assays

Mouse IFN-γ ELISPOT assays were performed using mouse IFN-γ ELISPOT kit (BD Biosciences, San Jose, CA), according to the manufacturer’s protocol. Briefly, 96-well ELISPOT plates were coated with IFN-γ capture antibody at 4 °C overnight, followed by blocking with culture medium (10% FCS-supplemented RPMI1640) for 1 h at room temperature. Lymphocytes were isolated from mouse allograft draining lymph nodes at 96-hour post-transplant by magnetic separation using the Pan T Cell Isolation Kit II (Miltenyi Biotec) or EasySep Mouse T Cell Isolation Kit (StemCell Technologies). 5 × 10^5^ T cells were incubated together with 5 × 10^5^ irradiated donor, host-derived or third-party splenocytes at 37 °C 5% CO_2_ for 48 h. T cells were also incubated with 5 × 10^5^ irradiated skin cells isolated from WT or Batf3^−/−^ allografts, transplanted 24 h before the cell culture setup at 37 °C 5% CO_2_ for 48 h. Alternatively, splenic T cells isolated from naive mice were incubated together with WT or March1^−/−^ BMDCs at 37 °C 5% CO_2_ for 24 h. Detected spots were counted using an ImmunoSpot analyzer (Cellular Technology).

Human IFN-γ ELISPOT assays were similarly performed as above, using human IFN-γ ELISPOT kit (R&D Systems). 5 × 10^5^ human peripheral blood mononuclear cells (PBMCs) were cultured together with 5 × 10^5^ irradiated human BMDCs prepared as above, at 37 °C 5% CO_2_ for 24 h.

### Mixed lymphocyte reaction

Human PBMCs were labeled with Cell Trace Violet (Invitrogen) and co-cultured with carboxyfluorescein succinimidyl ester (CFSE)-labeled BMDCs in RPMI with 10% human serum, l-glutamine and penicillin/streptomycin for 5 days at 37 °C, 5% CO_2_. Cell proliferation was analyzed by flow cytometry using FACS Canto II (BD Biosciences).

### Statistical analysis

Statistical analysis was performed using the software Prism5 (Graphpad Software Inc.). Differences between specific points were determined by the Student’s *t-*test, or when appropriated, the nonparametric Mann–Whitney. The one-way analysis of variance (ANOVA) test was used to determine differences between groups. Multiple comparisons among levels were checked with Tukey posthoc tests. To analyze graft survival and determine the MST, the Kaplan–Meier/log-rank test was used. The level of significance was set at *p* < 0.05. In all experiments, we used at least 3 samples per studied group.

### Study approval

All procedures were previously reviewed and approved by the Ethics Committee for the Use of Animals of Pontifícia Universidade Católica do Rio Grande do Sul (CEUA-PUCRS) under protocol ID CEUA 12/00316, Harvard Medical School IACUC 2016N000250 and Brigham and Women’s Hospital (BWH) IACUC 05050. For human studies, all subjects provided written informed consent to participate in the study, as approved by the Human Research Committee at PUCRS (#844.206). Human tissues were obtained via BWH Tissue Bank and via a clinical trial approved by the human research committee at Brigham and Women’s Hospital (2008BP00055).

### Data availability

The data supporting the findings of this study are available within the article and its Supplementary Information files or from the corresponding authors on reasonable request.

## Electronic supplementary material

Supplementary Information
