# Supplementary material for: March1-dependent modulation of donor MHC II on CD103+ dendritic cells mitigates alloimmunity
Source: Nat Commun. 2018 Aug 28;9:3482. doi: 10.1038/s41467-018-05572-z (PMC6113260; doi:10.1038/s41467-018-05572-z)
Supplement: Supplementary file 1 — Supplementary Information [file 41467_2018_5572_MOESM1_ESM.pdf]

March1-dependent modulation of donor MHC II on CD103$^+$ dendritic cells mitigates alloimmunity

Borges et al.

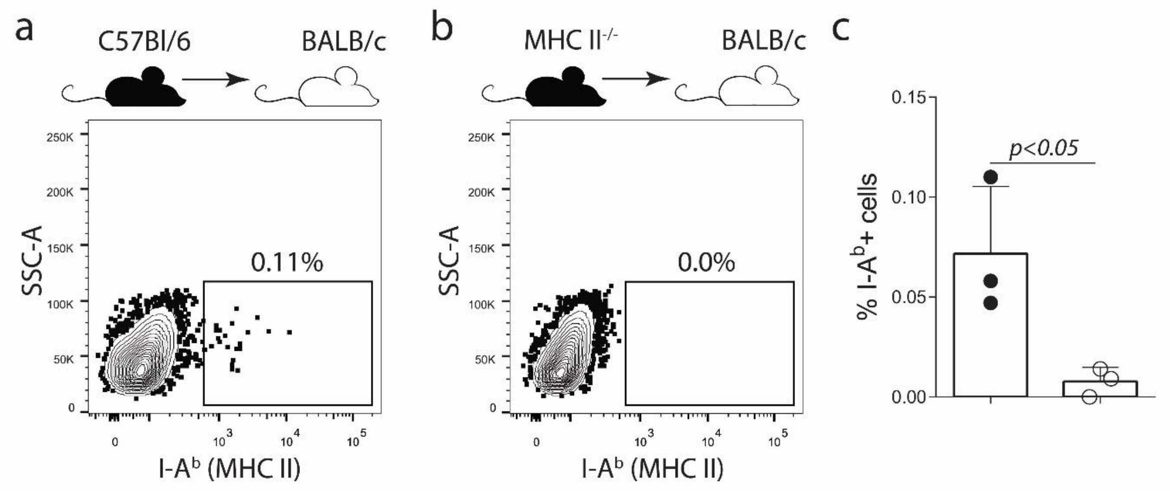

Supplemental Figure 1. Donor MHC II (I-A^{b}) from B6 MHC II$^{-/-}$ skin grafts are undetectable in host dLNs. Detection (**a, b**) and quantification (**c**) of I-A^{b+} events in dLN of BALB/c mice (H-2K^{d}/I-A^{d}) that received allogeneic WT or MHC II$^{-/-}$ skin grafts (B6 background, H-2K^{b}/I-A^{b}), at 24h post-transplant (n=3 mice/group). Data are represented as mean ± S.D. Statistics using *t* test. Representative results of two independent experiments.

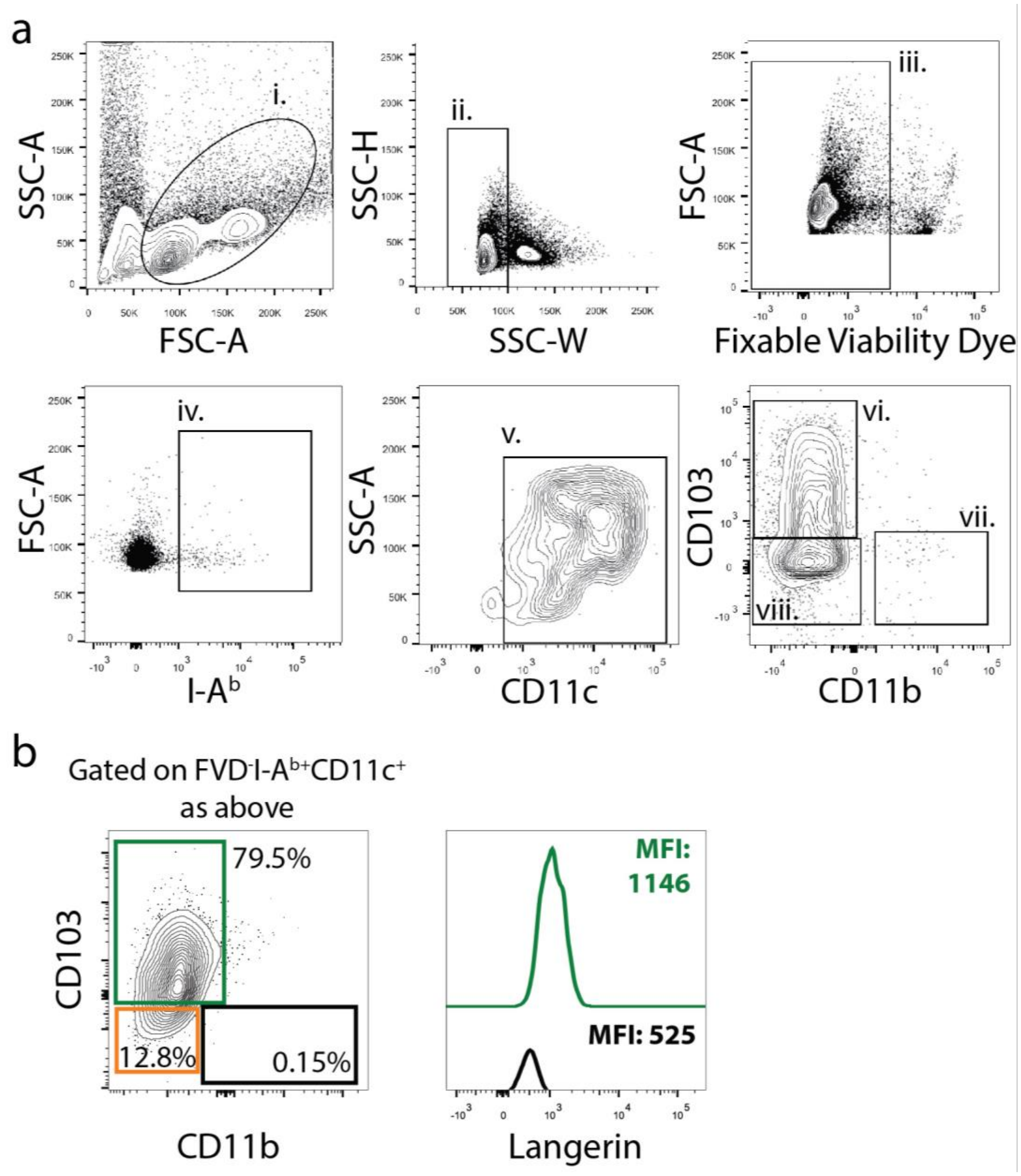

Supplemental Figure 2. Gating strategy for the detection of donor MHC II-expressing dLN DC subsets after fully mismatch murine skin transplantation. (**a**) Detection of I-A^{b+} DC subsets in dLN of BALB/c mice (H-2K^{d}/I-A^{d}) that received allogeneic B6 skin grafts (H-2K^{b}/I-A^{b}). (**b**) Alternative strategy to demonstrate that DCs carrying donor MHC II are CD11c^{+}CD11b^{-}CD103^{+} Langerin^{+}.

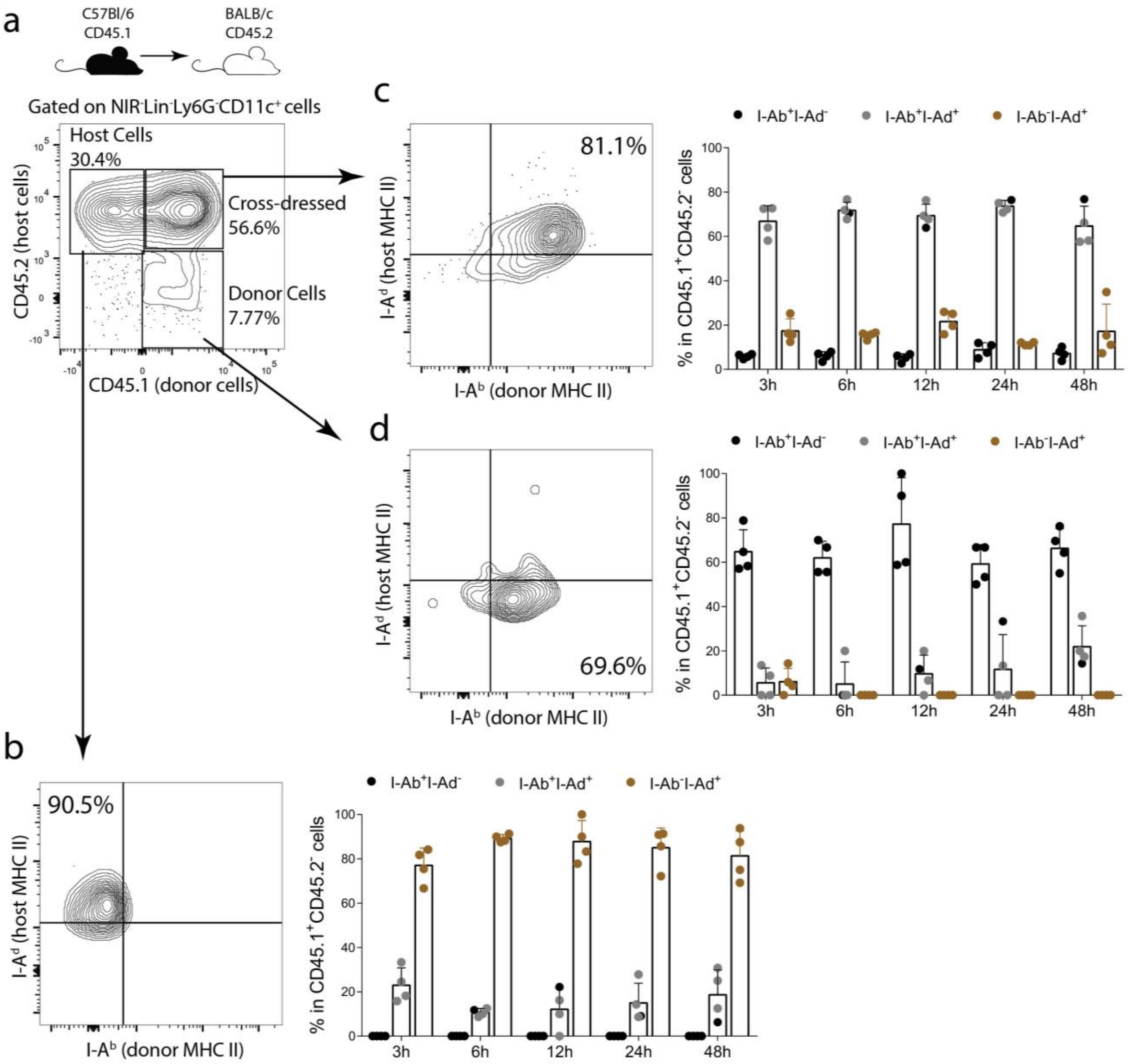

Supplemental Figure 3. Expression of I-A^{d} and I-A^{b} on host, cross-dressed and donor cells. CD45.1 (B6 - H-2K^{b}/I-A^{b}) skin grafts were transplanted to CD45.2 BALB/c (H-2K^{d}/I-A^{d}) mice. (**a**) Detection of donor (CD45.1^{+}CD45.2^{-}), host (CD45.1^{-}CD45.2^{+}) and cross-dressed host (CD45.1^{+}CD45.2^{+}) DCs in host dLN by flow cytometry at 24h post-transplant. Expression of I-A^{d} and/or I-A^{b} on (**b**) host CD45.1^{-}CD45.2^{+} cells, (**c**) cross-dressed host CD45.1^{+}CD45.2^{+} cells and (**d**) donor CD45.1^{+}CD45.2^{-} cells at 3, 6, 12, 24 or 48h post-transplant. Data are represented as mean ± S.D. n=3-4 mice per time-point/group**.** Representative results of two independent experiments.

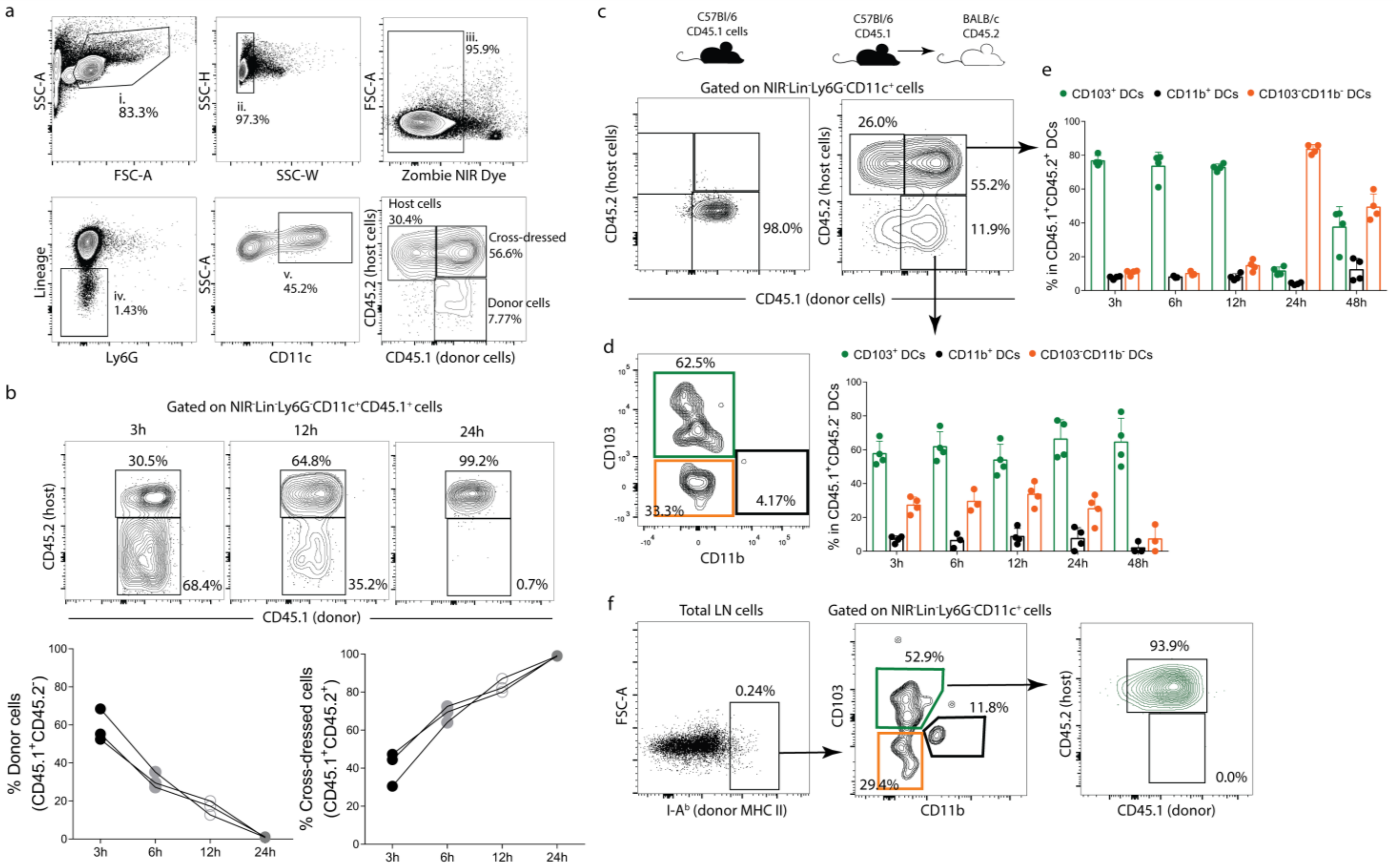

Supplemental Figure 4. Strategy for detection of donor and host DCs using different congenic markers. CD45.1 (B6 - H-2K^b/I-A^b) skin grafts were transplanted to CD45.2 BALB/c (H-2K^d/I-A^d) mice. (**a**) Gating strategy for the detection of donor (CD45.1$^+$CD45.2$^-$), host (CD45.1$^-$CD45.2$^+$) and cross-dressed host (CD45.1$^+$CD45.2$^+$) DCs in host dLN by flow cytometry at 24h post-transplant. (**b**) Analysis of donor-derived (CD45.1$^+$CD45.2$^-$) and host cross-dressed (CD45.1$^+$CD45.2$^+$) cells in CD45.1$^+$ cells at 3h, 6h, 12h and 24h after the transplant. Each line represents one mouse. (**c**) Detection of donor (CD45.1$^+$CD45.2$^-$), host (CD45.1$^-$CD45.2$^+$) and cross-dressed host (CD45.1$^+$CD45.2$^+$) DCs in host dLN by flow cytometry at 24h post-transplant. Detection and quantification CD103$^+$CD11b$^-$, CD103$^-$CD11b$^+$ and CD103$^-$CD11b$^-$ DC subsets in (**d**) CD45.1$^+$CD45.2$^-$ (donor) or (**e**) CD45.1$^+$CD45.2$^+$ (host cross-dressed) cells over time. (**f**) Draining-LN CD103$^+$ DCs expressing I-A^b (donor MHC II) are all host cross-dressed (CD45.1$^+$CD45.2$^+$) DCs at 24h post-transplant. Data are represented as mean ± S.D. (n=3-4 mice per time-point/group). All data are representative results of two independent experiments.

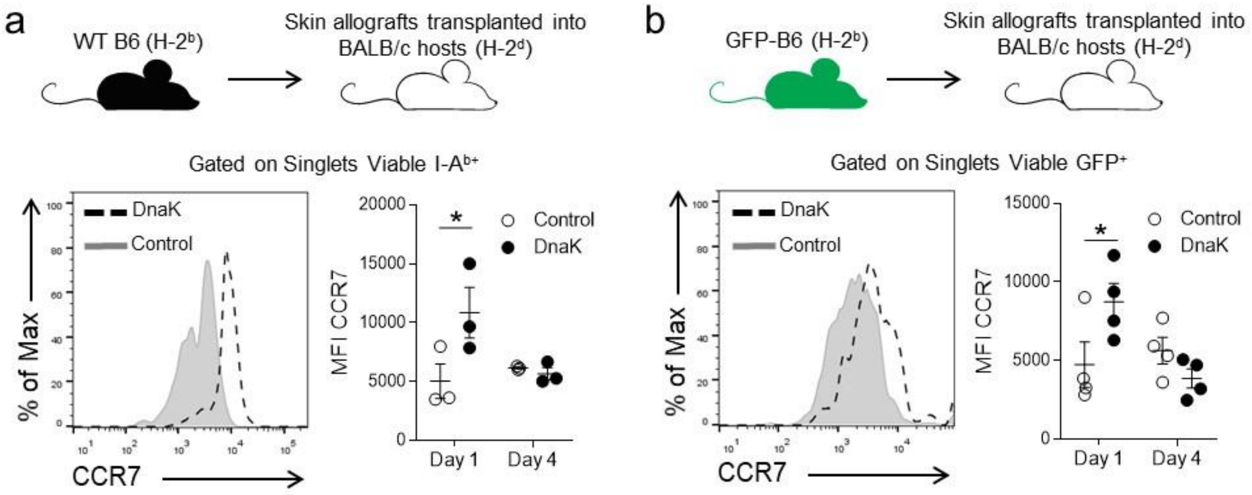

Supplemental Figure 5. DnaK treatment does not affect the migration of donor cells to draining lymph nodes. Skin allografts from B6 or Actin-GFP mice were immersed in PBS solution with or without DnaK for 1h, followed by transplantation to fully MHC mismatched BALB/c hosts. Mean fluorescence intensity (MFI) of CCR7 in (**a**) I-A^{b+} or (**b**) GFP^{+} population. Representative of three experiments. Statistics using ANOVA with Tukey post-test (n=3-4 mice per time-point/group); *$p<0.05$. Data are represented as mean ± S.D.

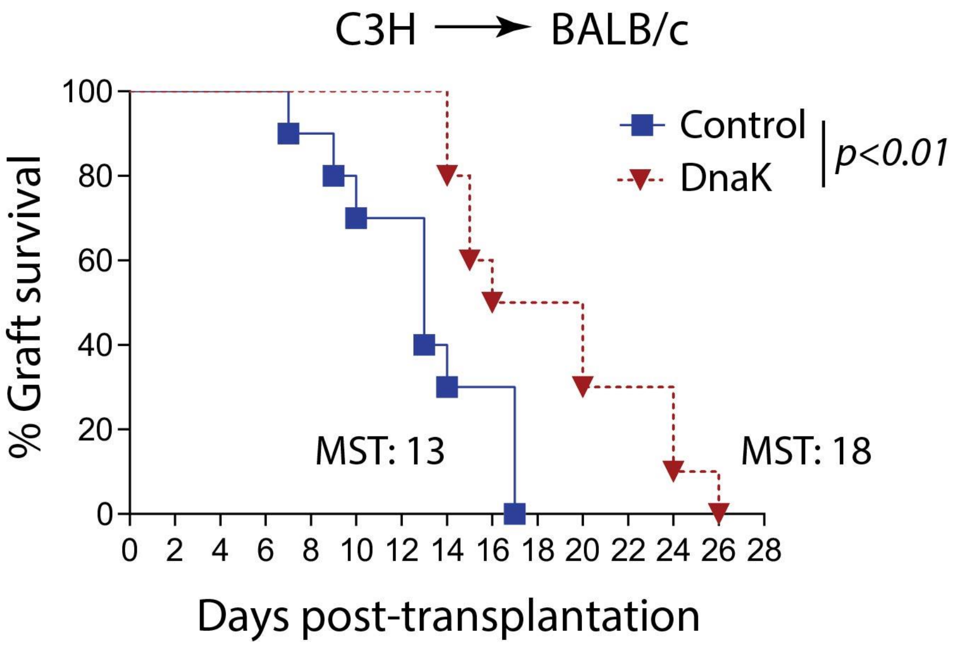

Supplemental Figure 6. In situ treatment with DnaK prior to skin transplant prolongs allograft survival. Skin allografts from C3H mice (H-2K^k/I-A^k) were immersed in PBS solution with or without DnaK for 1h, followed by transplantation into fully MHC mismatched BALB/c hosts (H-2K^d/I-A^d). Percent of graft survival after DnaK treatment (n= 10/group). Mean survival time (MST). Statistic by long-rank test. Results are pooled from two experiments.

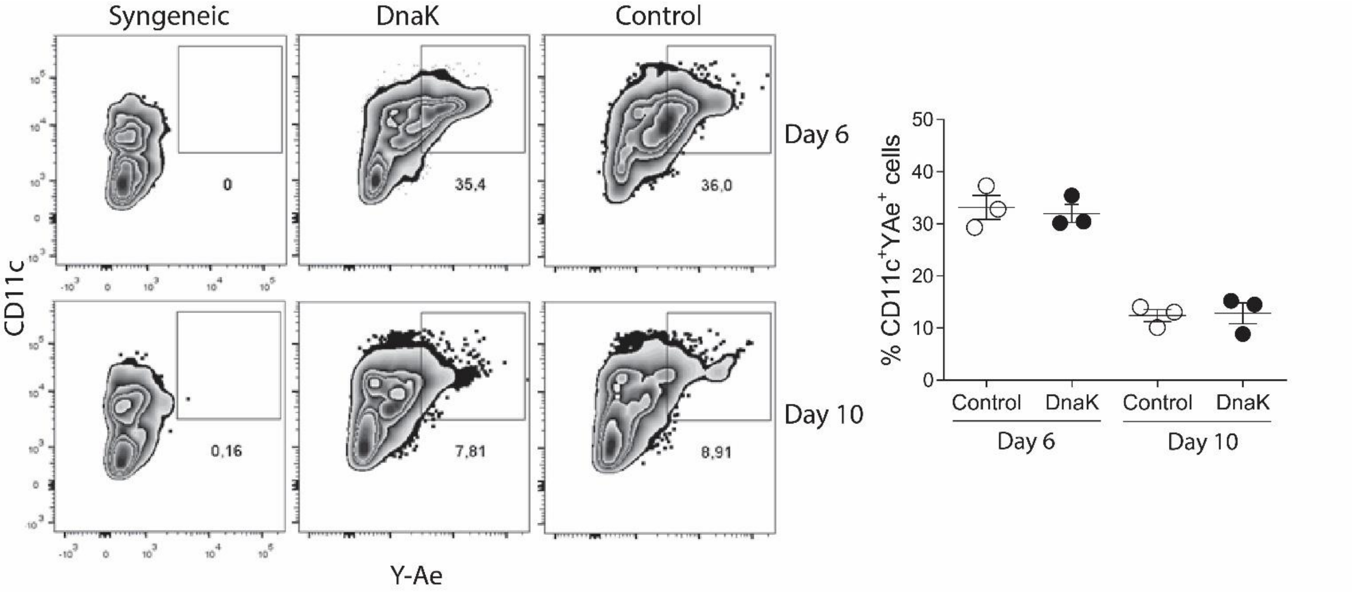

Supplemental Figure 7. DnaK treatment does not affect indirect pathway of allorecognition. Skin allografts from BALB/c mice (H-2K^d/I-A^d) were transplanted to fully MHC-mismatched B6 (H-2K^b/I-A^b) or BALB/c syngeneic hosts. Host dLN DCs were analyzed by their capacity to present a donor-derived peptide (E$\alpha_{52\text{-}68}$) to I-A^b context (host MHC II), using the Y-Ae antibody. Data are represented as mean ± S.D. n=3 mice per time-point/group. Representative results of two independent experiments.

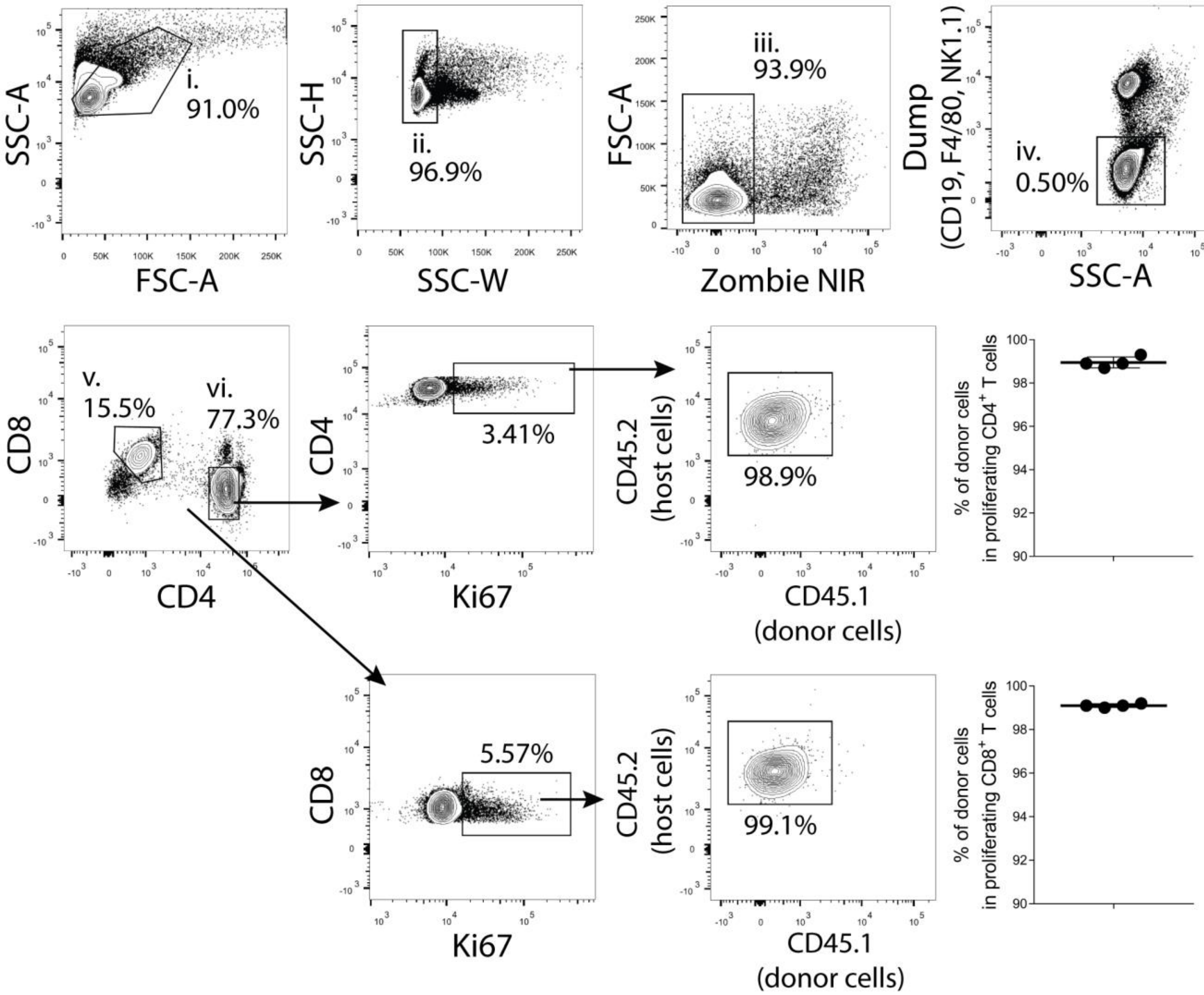

Supplemental Figure 8. Proliferating T cells are from host origin. CD45.1 (B6 - H-2K^b/I-A^b) skin grafts were transplanted to CD45.2 BALB/c (H-2K^d/I-A^d) mice. Gating strategy for the detection of proliferating (Ki67$^+$) CD4$^+$ or CD8$^+$ T cells expressing donor (CD45.1$^+$CD45.2$^-$), host (CD45.1$^-$CD45.2$^+$) or host cross-dressed (CD45.1$^+$CD45.2$^+$) markers. Both CD4$^+$Ki67$^+$ and CD8$^+$Ki67$^+$ cell populations were from host (CD45.1$^-$CD45.2$^+$). Data are represented as mean ± S.D. n=4 mice per time-point/group. Representative results of two independent experiments.

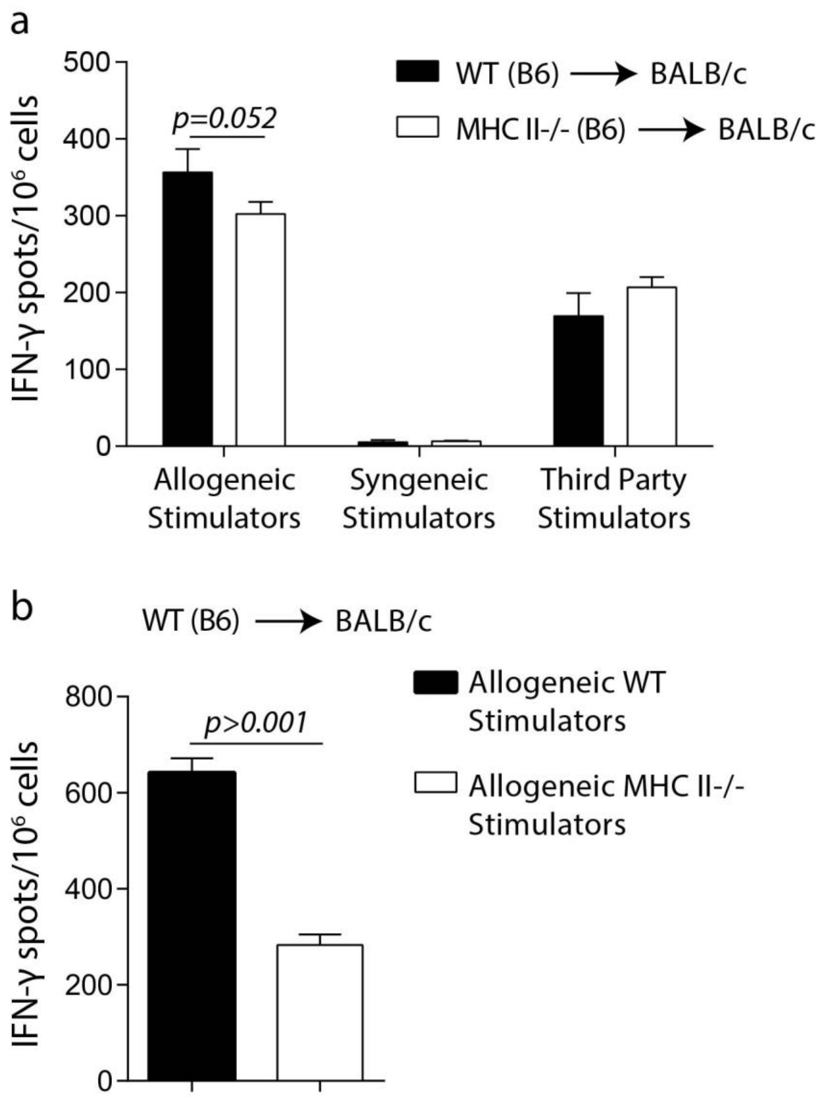

Supplemental Figure 9. Contribution of donor MHC II to allo-T cell responses in skin graft rejection model. (**a**) WT or MHC $II^{-/-}$ skin grafts (B6 background, H-$2K^b$/I-A^b) were transplanted into BALB/c (H-$2K^d$/I-A^d) hosts. At 96h after the transplant, T cells from allograft dLN were magnetically isolated and cultured with allogeneic (B6), syngeneic (BALB/c) or third-party (C3H, H-$2K^k$/I-A^k) irradiated stimulators (splenocytes) for 48h *in vitro* in mouse IFN-γ-coated plates. Mean numbers of spots per million T cells ± S.D. in triplicates and pooled from three mice per group. Statistics using ANOVA with Tukey post-test. Representative results of two independent experiments. (**b**) WT skin grafts (B6 background) were transplanted into BALB/c hosts. At 96h after the transplant, T cells from allograft dLN were magnetically isolated and cultured with allogeneic (B6) WT or MHC $II^{-/-}$ irradiated stimulators (splenocytes) for 48h *in vitro* in mouse IFN-γ-coated plates. IFN-γ production by T cells was measured by ELISPOT. Mean numbers of spots per million T cells ± S.D. in triplicates and pooled from three mice per group. Statistics using t test. Representative results of two independent experiments.

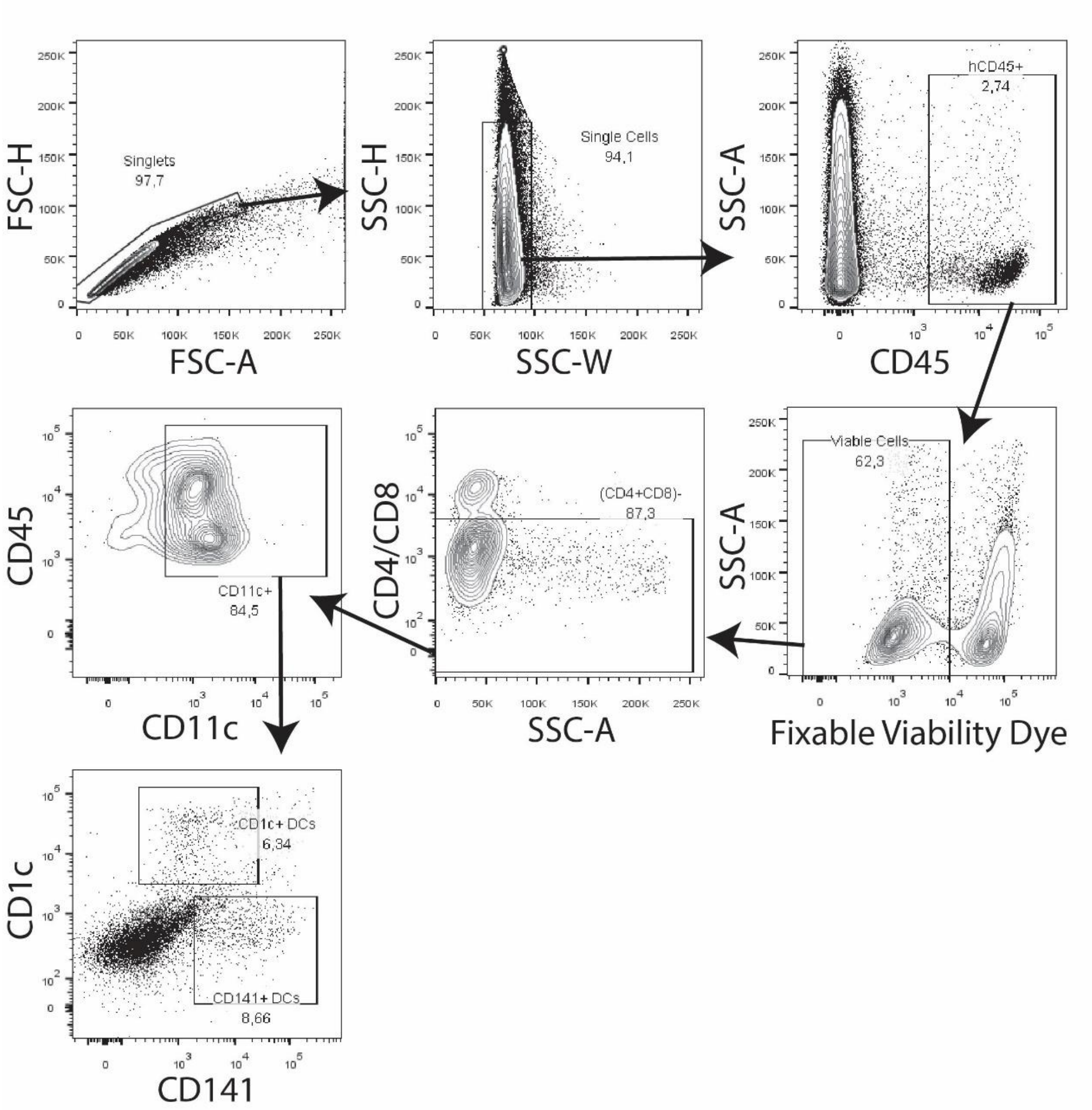

Supplemental Figure 10. Gating strategy used in the analysis of human skin-resident dendritic cells. Healthy human skin cells were isolated, stained for CD45, Fixable Viability Dye, CD4, CD8, CD11c, CD1c, CD141 and HLA-DR, and analyzed by flow cytometry.

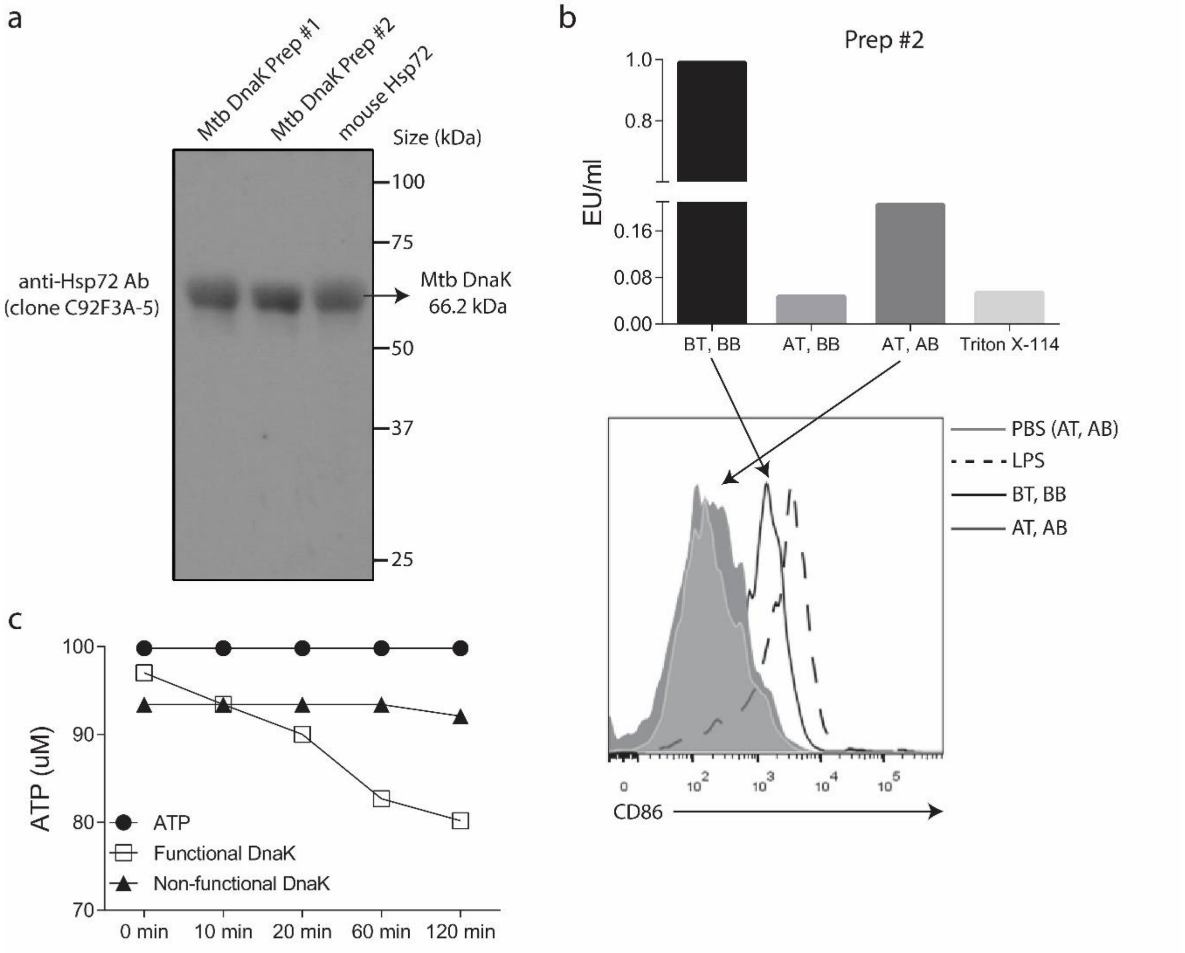

Supplemental Figure 11. DnaK purification quality controls. (**a**) Purified DnaK was detected using Western blot. (**b**, upper) Endotoxin levels of DnaK preparation at all purification stages: BT, BB (before Triton, before beads incubation); AT, BB (after Triton, before beads); AT, AB (after Triton and after beads incubation) or only Triton X-114 as a control. (**b**, lower) B6 mice were intravenously injected with LPS (25 µg), Bio-beads-treated PBS 1x or DnaK with or without Triton X-114 treatment (both 30 µg). After 6h, mice were sacrificed and CD86 expression was analyzed in splenic $CD11c^+$ cells by flow cytometry. (**c**) Evaluation of ATP hydrolysis by functional DnaK or non-functional DnaK (freeze-thawing). Proteins (5 µg) were incubated with 100 µM ATP and ATP amounts were analyzed by HPLC at 0, 10, 20, 60 and 120 min after treatment. Results are expressed as total amount of the ATP (µM) in respective incubation time. Data are represented as mean. Representative results of at least two independent experiments.
